# Supplementary material for: Rationally designed synthetic protein hydrogels with predictable mechanical properties
Source: Nat Commun. 2018 Feb 12;9:620. doi: 10.1038/s41467-018-02917-6 (PMC5809592; doi:10.1038/s41467-018-02917-6)
Supplement: Supplementary file 1 — Supplementary Information [file 41467_2018_2917_MOESM1_ESM.pdf]

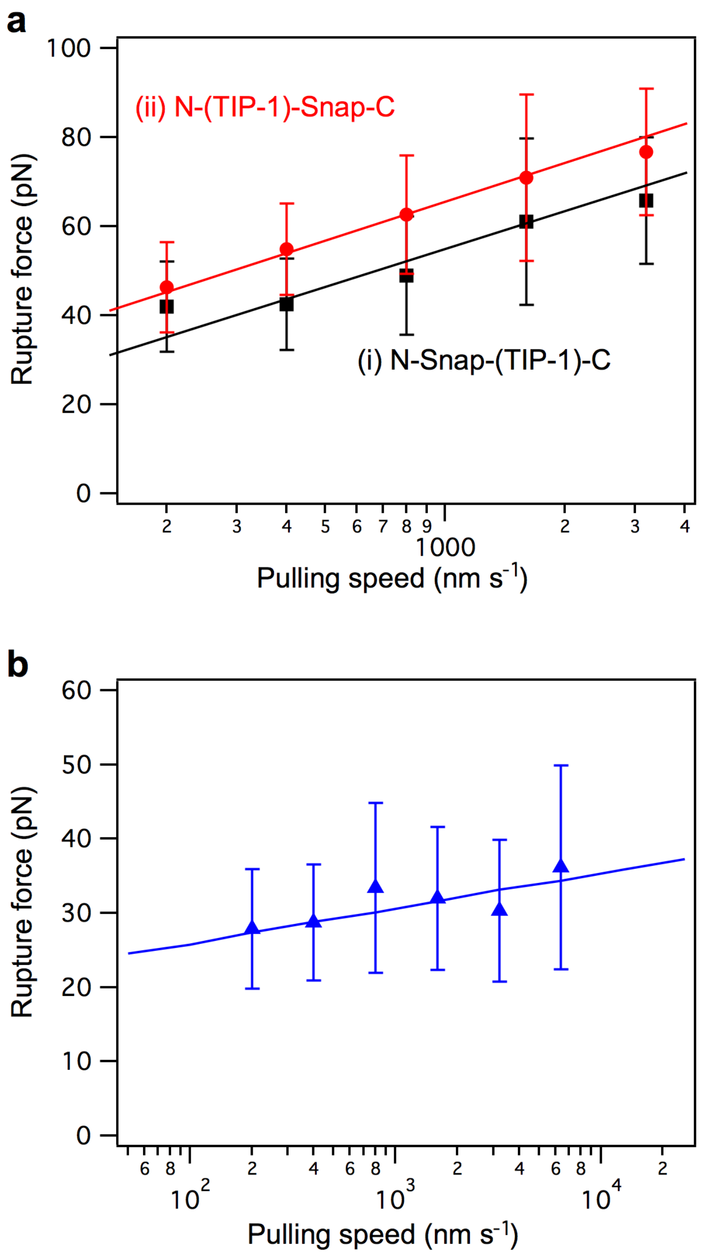

Supplementary Figure 1 Loading rate dependent experiments. a) The rupture forces of Tip-1:Kir complexes from two pulling directions. b) the mechanical unfolding of SH3.

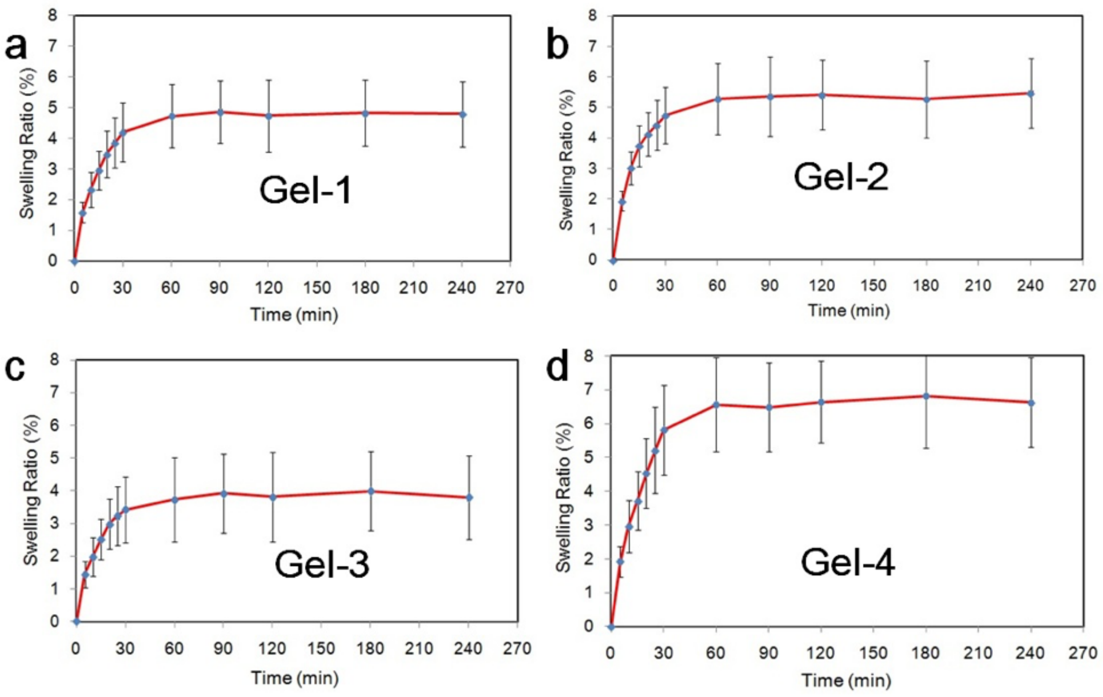

Supplementary Figure 2 Swelling ratios of Gel 1–4 (180 mg mL^{-1}) in PBS buffer at room temperature. a) Gel-1, b) Gel-2, c) Gel-3, and d) Gel-4. The ring-shaped hydrogels were weighted immediately after being taken out of the molds, and the weight was recorded as W_0. Then the hydrogels were soaked in PBS, pH 7.4, at the room temperature. After certain time, the hydrogel rings were taken out of PBS buffer, blotted onto tissue paper to remove excess buffer and weighted as W_t. The swelling ratio was calculated according to the formula: Swelling ratio (%)＝ $(W_t - W_0)/ W_0]\times100\%$. Two different samples were measured and the average value was reported. The error bars represent standard deviation.

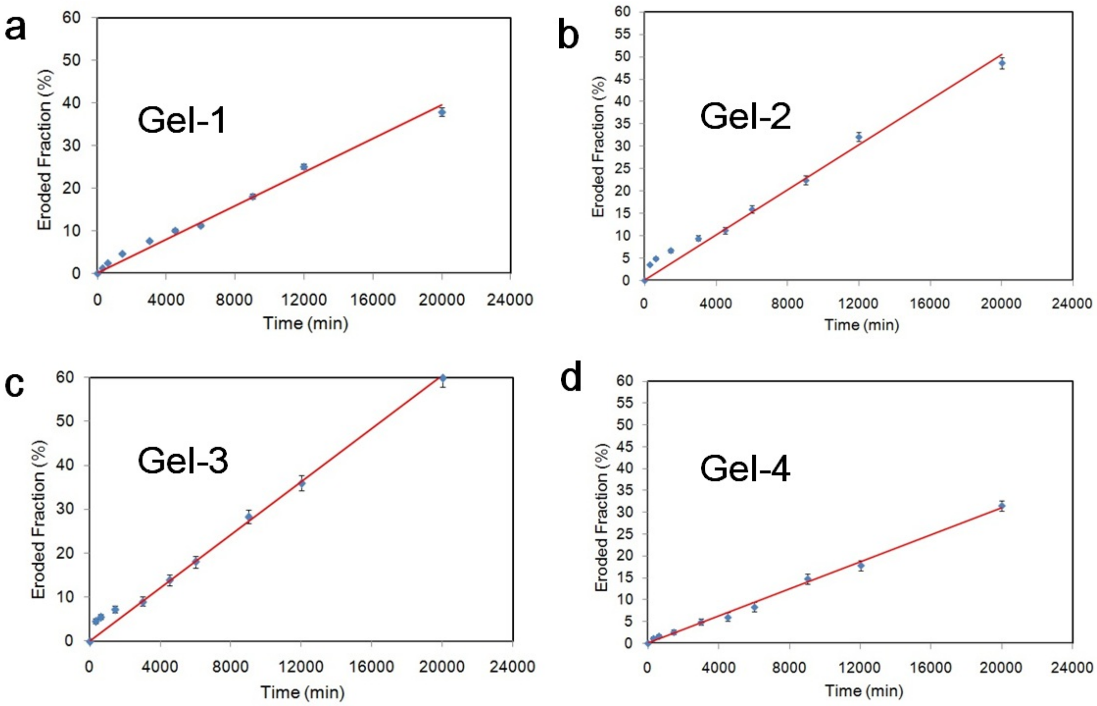

Supplementary Figure 3 Erosion profiles of 100 mg of Gel 1–4 at room temperature in 100 mM phosphate buffer, pH 7.4. All hydrogels (at 180 mg mL^{-1}) were with a surface area of 0.86 cm^2. The erosion was too slow to be reliably determined using UV spectra if the gel samples were kept still in solution. Therefore, the erosion was measured under mild mechanical shaking. In the experiment, 100 mg of hydrogel was transferred into a cylindrical glass tube with a flat bottom (1.05cm diameter). The glass tube with the hydrogel was then centrifuged at 1700 g for 10 minutes to completely flat down hydrogel sample to the bottom and smooth the surface of the hydrogel. The hydrogel was allowed to stand overnight. The thin gel film together with the glass tube was then soaked in 5 mL of 100 mM phosphate buffer, pH 7.4, in a scintillation vial. The whole setup was placed on a compact rocker tilting at 50 rpm with amplitude of ±9°, at room temperature. The erosion profiles were determined by measuring the protein absorbance at 280 nm of the supernatant at successive time points using Nano-drop ultraviolet-visible spectrophotometer. Two different samples were measured and the average value was reported. Error bars represent standard deviation of the experimental data. a) Erosion profile of Gel-1. A

linear regression (solid line) measures an erosion rate of 3.55×10^{-4} mg cm^{-2} min^{-1}. b) Erosion profile of Gel-2. A linear regression (solid line) measures an erosion rate of 4.54×10^{-4} mg cm^{-2} min^{-1}. c) Erosion profile of Gel-3. A linear regression (solid line) measures an erosion rate of 4.76×10^{-4} mg cm^{-2} min^{-1}. d) Erosion profile of Gel-4. A linear regression (solid line) measures an erosion rate of 2.79×10^{-4} mg cm^{-2} min^{-1}.

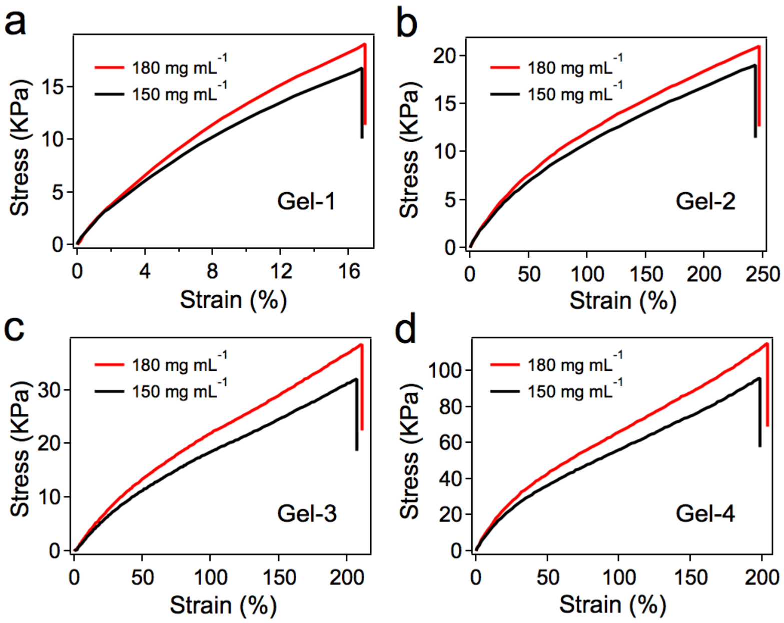

Supplementary Figure 4 The stress-strain curves for Gel-1 (a), Gel-2 (b), Gel-3 (c), and Gel-4 (d) at two different concentrations (150 and 180 mg mL^{-1}). The hydrogel was stretched until break at a constant strain rate of ~ 20 mm min^{-1}. The minimal gelation concentrations were determined to be ~ 100 mg mL^{-1} by the vial inversion method. But when the concentrations are below 140 mg mL^{-1}, the ring-shaped hydrogels are not strong enough to be taken out from the mold. On the other hand, the maximum solubility of the proteins is ~180-190 mg mL^{-1}. Therefore, we have only a very narrow range to study the effect of protein concentrations on the mechanical properties.

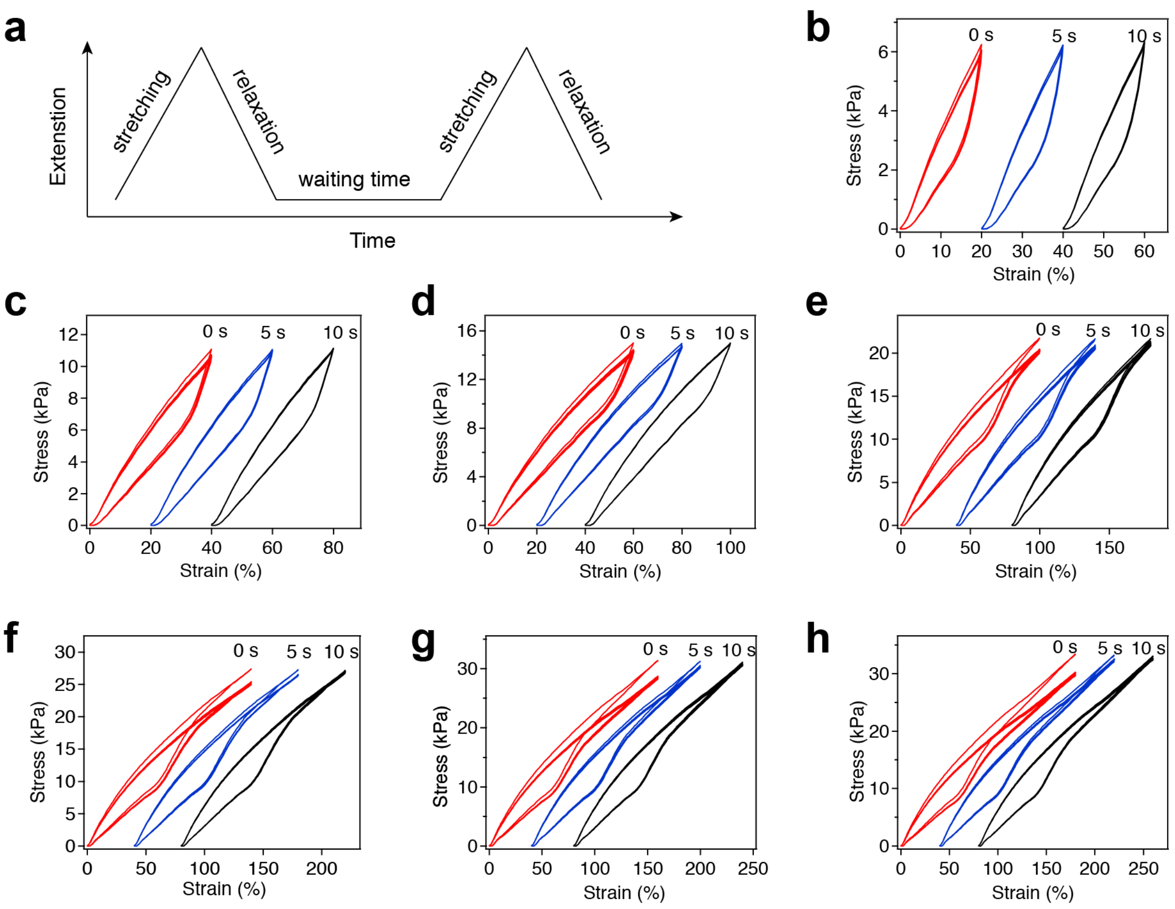

Supplementary Figure 5 The stretching-relaxation cycles of Gel-3 with different strain and different waiting time. a) The experimental scheme. The hydrogel (Gel-3) was stretched to the given length (20%, 40%, 60%, 100%, 140%, 160%, 180% strain, respectively), and then immediately relaxed to 0% strain. There are totally 5 continuous cycles with indicated waiting time between each cycle. All the experiments were at a constant strain rate of ~ 20 mm/min. b-h) The stress-strain curves for Gel-3 in different stretching-relaxation cycles with waiting time of 0, 5, and 10 s. The curves are horizontally offset for clarity.

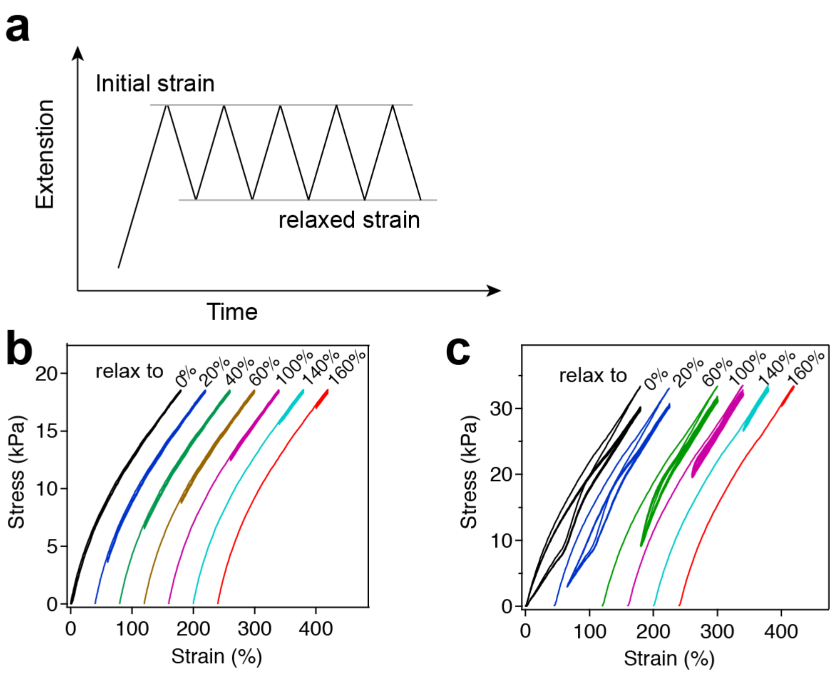

Supplementary Figure 6 The stretching-relaxation cycles of Gel-2 and Gel-3 at different residual strain. a) The experimental protocol. The hydrogel (Gel-2 or Gel-3) was stretched to 180% strain. Immediately after that, the hydrogel was relaxed to the given residual strain. There are totally 5 continuous cycles without any waiting time between each cycle. All the experiments were at a constant strain rate of 20 mm min^{-1}. b) The stress-strain curves for Gel-2. c) The stress-strain curves for Gel-3. The curves in b) and c) are offset for clarity.

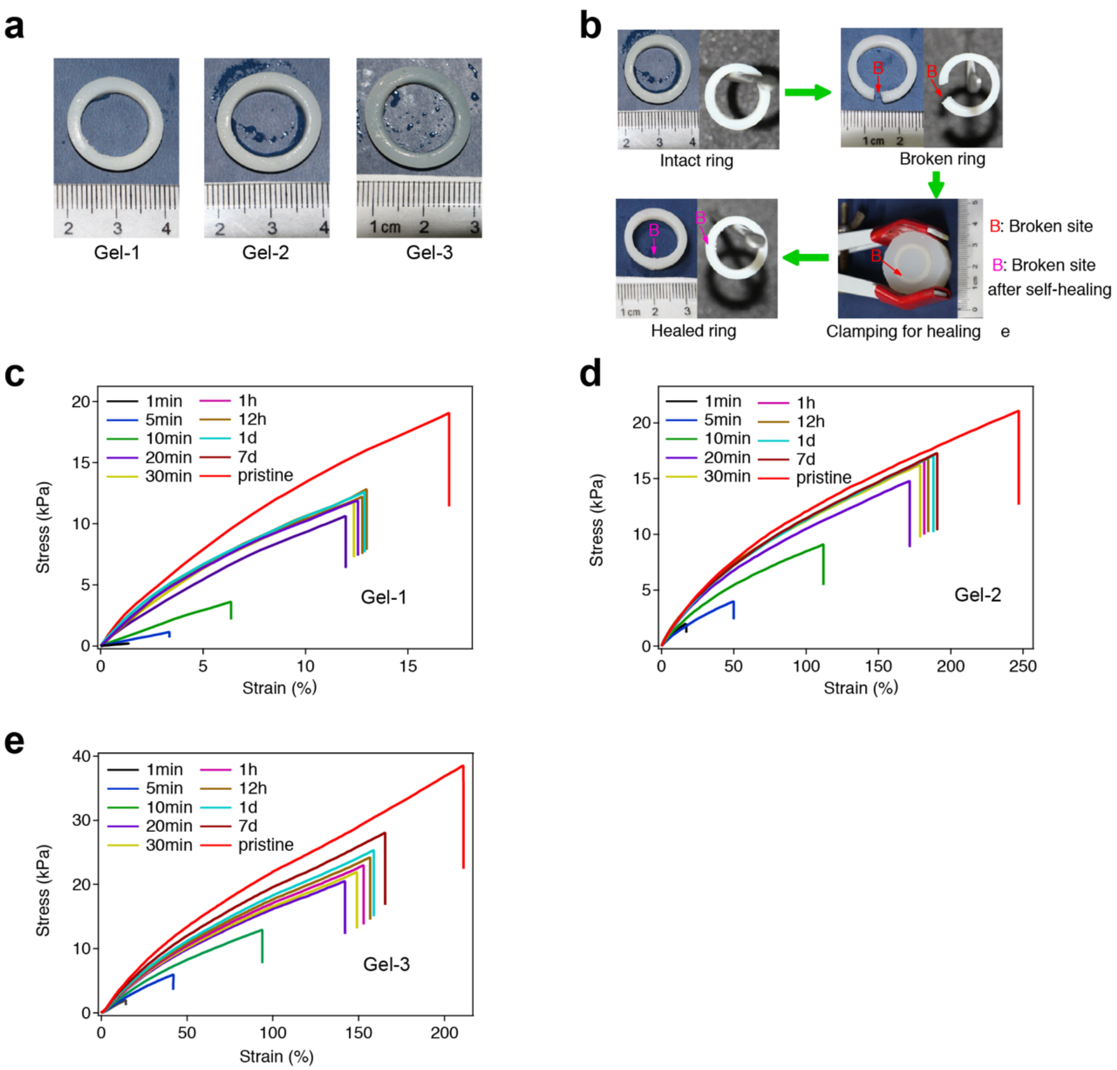

Supplementary Figure 7 The pictures and self-healing properties of Gel-1, Gel-2 and Gel-3. a) Representative gel pictures for Gel-1, Gel-2 and Gel-3 after equilibrium in PBS buffer for 1 day. b) General procedure for the self-healing test. First, the hydrogel ring was completely cut to produce a broken ring. Then, the broken ring was put back to the mold and clamped to move two broken interfaces together for different healing time. After healing, the hydrogel ring became intact and free-standing. The stress-strain curves for the hydrogel rings healed for different time was shown in c, d, and e. The pristine or the self-healed hydrogel was stretched until break at a constant strain rate of 20 mm min^{-1}.

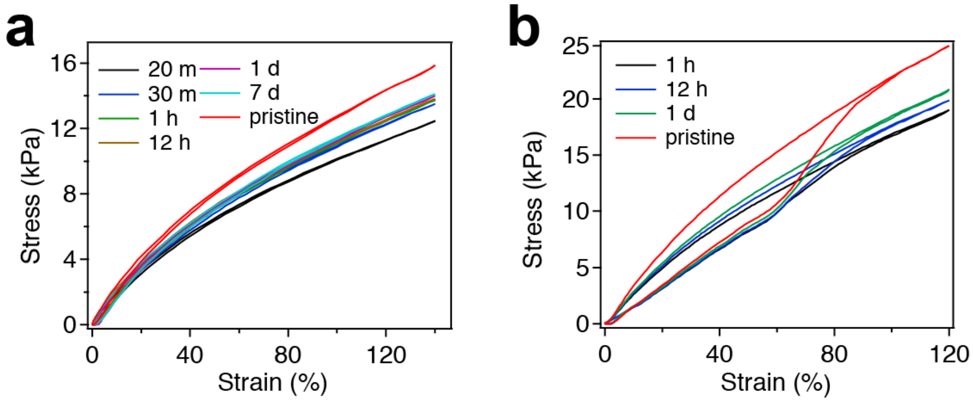

Supplementary Figure 8 The stretching-relaxation cycles of Gel-2 and Gel-3 after self-healing for different time periods. All the experiments were at a constant strain rate of ~ 20 mm min^{-1}. a) The stress-strain curves for Gel-2. The pristine or the self-healed hydrogel was stretched to 140% strain, and then immediately relaxed to 0% strain. b) The stress-strain curves for Gel-3. The pristine or the self-healed hydrogel was stretched to 120% strain, and then immediately relaxed to 0% strain. Because the self-healed Gel-1 can only be stretched to a strain of less than 15%, the stress-strain curves after self-healing were not measured.

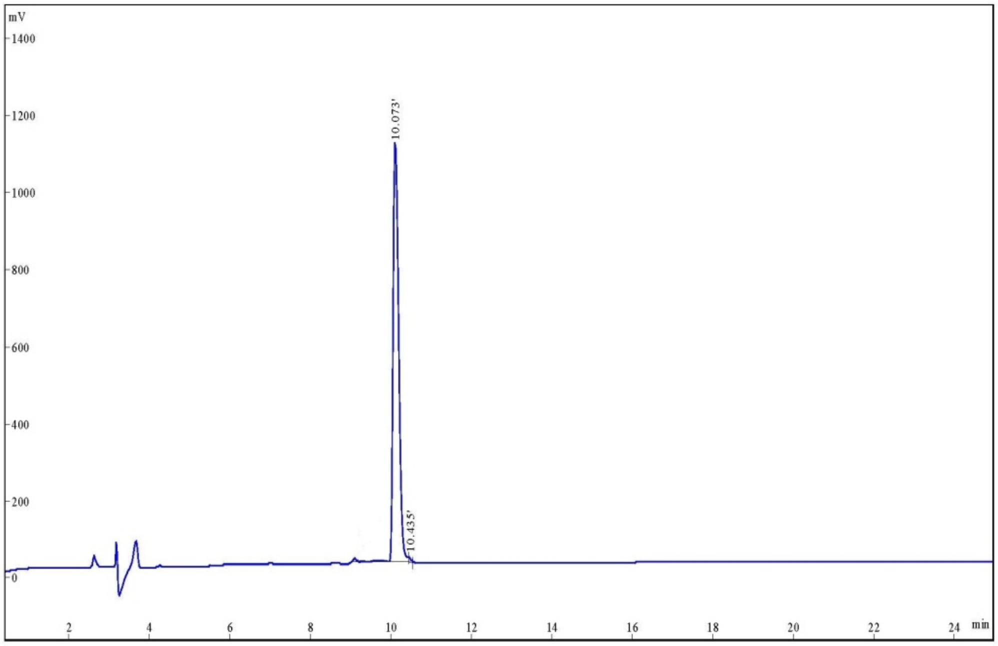

Supplementary Figure 9 HPLC chromatogram of Kir peptide (CNISYWRESAI).

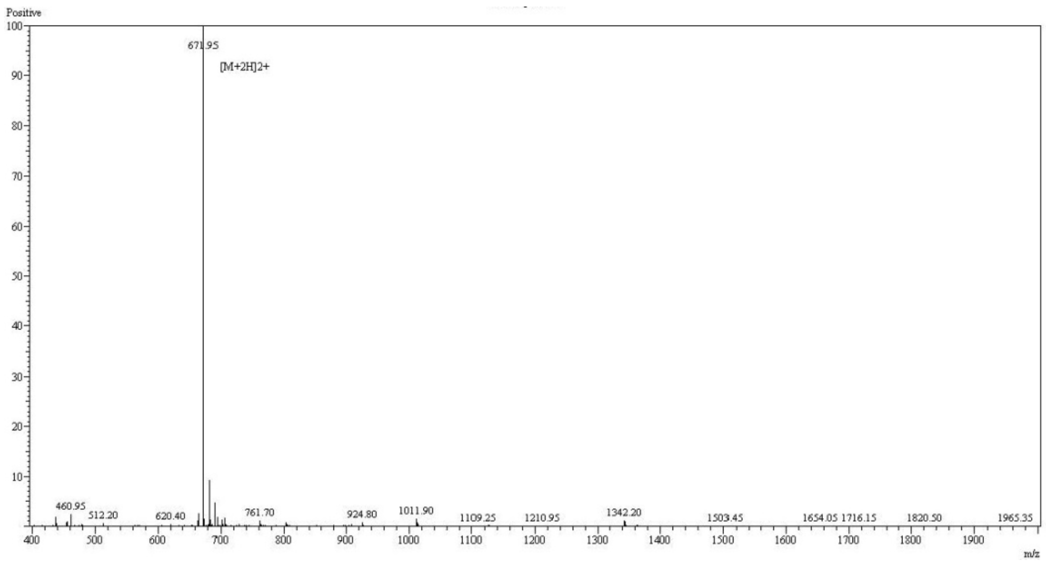

Supplementary Figure 10 Mass Spectroscopy of Kir peptide (CNISYWRESAI).

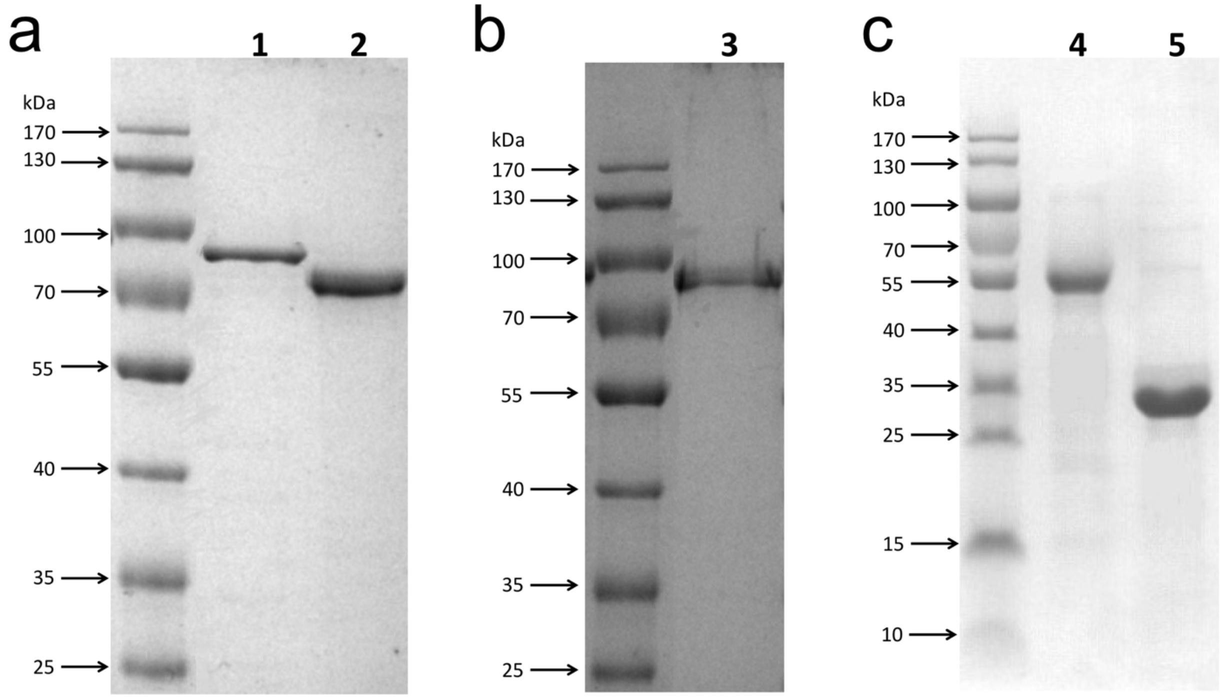

Supplementary Figure 11 Coomassie blue stained SDS-PAGE photograph for the polyproteins. a) TIP-1-$(GB1)_8$- TIP-1 (Lane 2), TIP-1-$(GB1\text{-}Hp67)_4$- TIP-1 (Lane 1). b) TIP-1-$(SH3)_8$- TIP-1 (Lane 3). c) Coh-$(GB1)_4$-cys (Lane 4) and cys-Xmod-Doc (Lane 5).

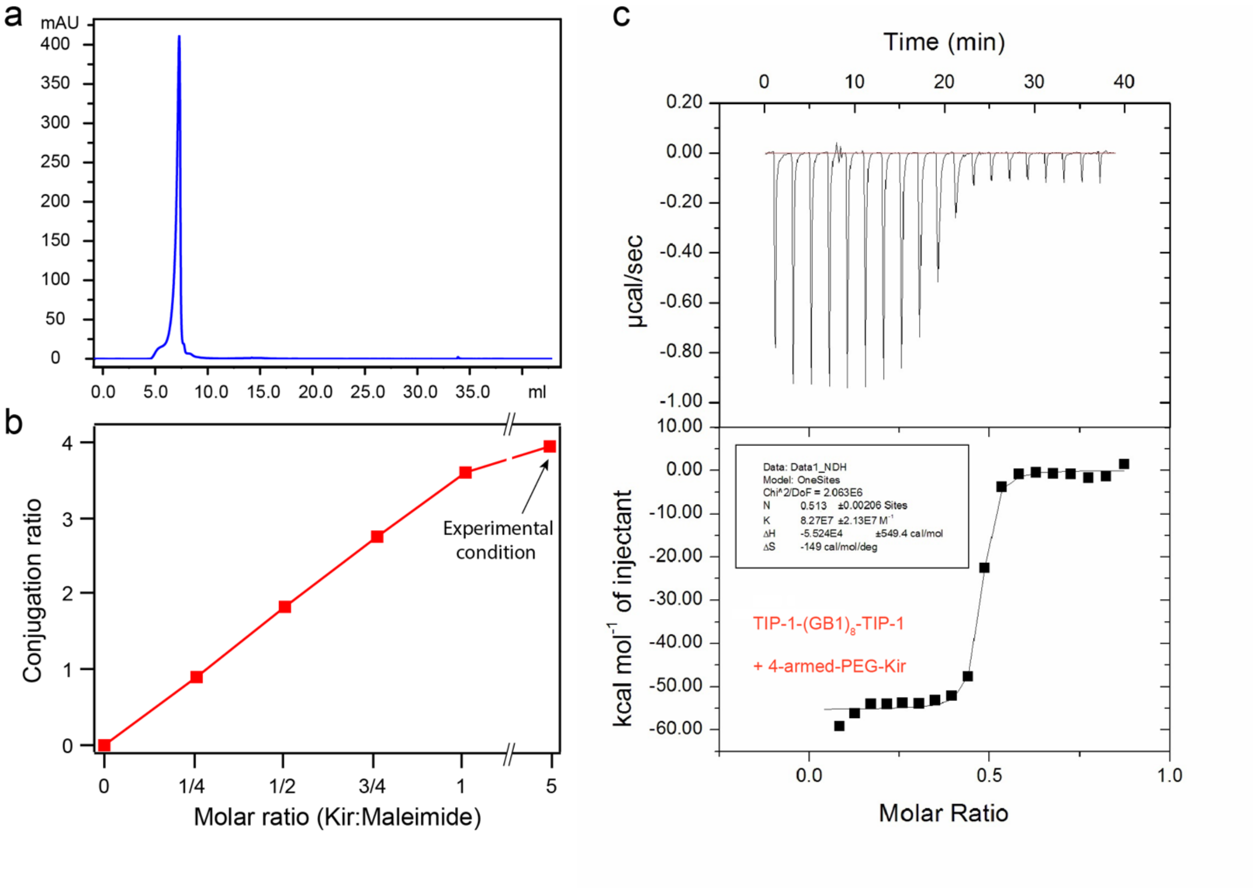

Supplementary Figure 12 Characterization of MCL of 4-armed PEG-Kir. a) HPLC trace of the MCL indicates the high purity of the purified MCL sample. b) The conjugation ratios of 4-armed PEG-maleimide after reacting with different molar ratios of kir peptides as determined by UV-absorbance at 280 nm during dialysis. The conjugation ratio was ~ 3.9 at the experimental conditions used for preparing MCL. c) The conjugation ratio of the MCL was further determined using ITC by titrating the protein solution of TIP-1-$(GB1)_4$-TIP-1 with 4-armed PEG-Kir. The conjugation ratio of Kir to 4-armed PEG was estimated to be ~3.90 (2/0.513), which was consistent with that determined by UV-Vis absorbance. The dissociation constant was estimated to be ~1.21 μM.

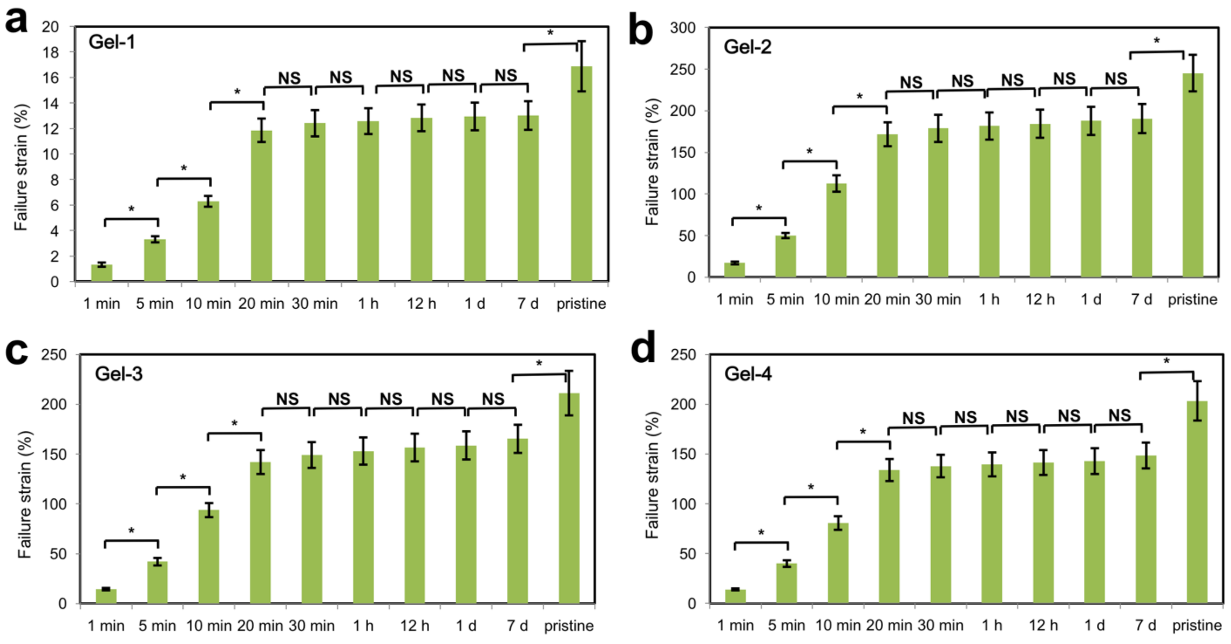

Supplementary Figure 13 Failure strains of the pristine and the various time self-healed hydrogels. a) Gel-1, b) Gel-2, c) Gel-3, and d) Gel-4. The hydrogel was stretched until break (failure) at a constant strain rate of 20 mm min^{-1}. Student's t-test was used for statistical analysis. When p value is below 0.05, it is considered being statistically significant. NS, not significant, *P < 0.05. The time point after which the recovery of the mechanical properties is not significant (P < 0.05) is defined as the recovery time. Based on this definition, the recovery time for all gels was ~ 20 min.

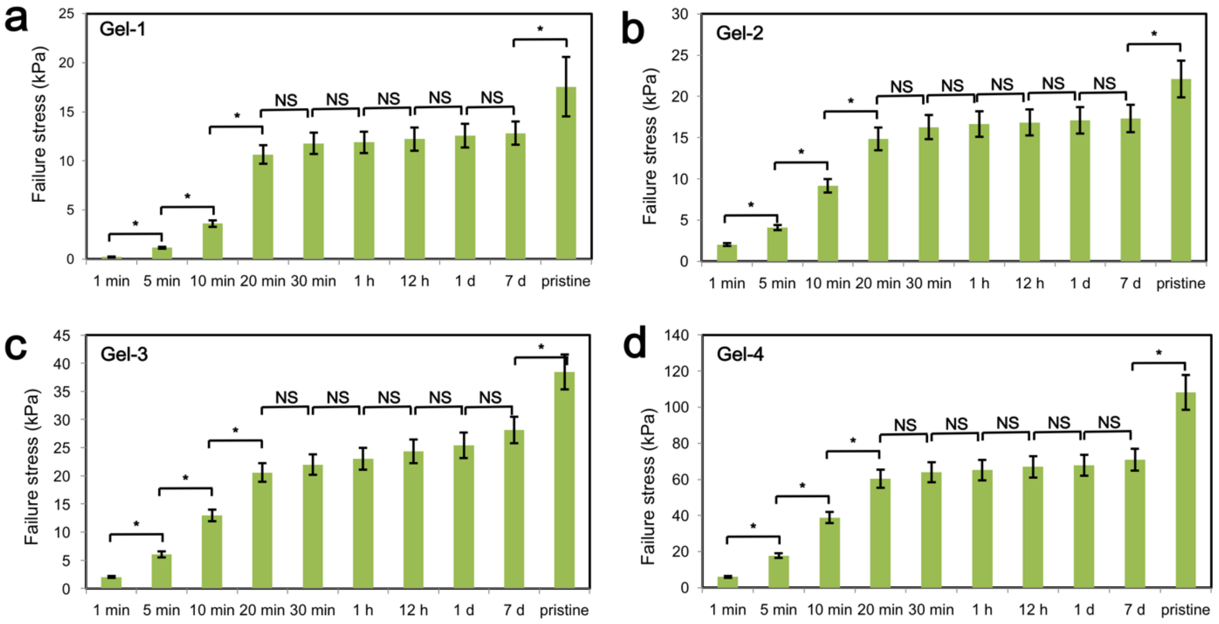

Supplementary Figure 14 Failure stress of the pristine and the various time self-healed hydrogels. a) Gel-1, b) Gel-2, c) Gel-3, and d) Gel-4. The hydrogel was stretched until break (failure) at a constant strain rate of 20 mm min^{-1}. Student's t-test was used for statistical analyses. When p value is below 0.05, it is considered being statistically significant. NS, not significant, **P* < 0.05. The time point after which the recovery of the mechanical properties is not significant (P < 0.05) is defined as the recovery time. Based on this definition, the recovery time for all gels was ~ 20 min.

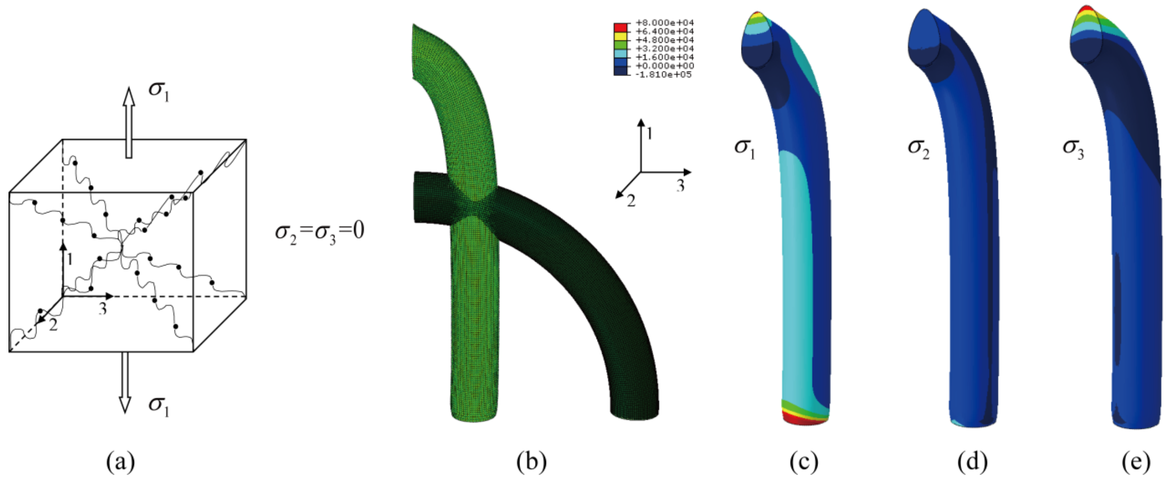

Supplementary Figure 15 Schematic of the RVE under uniaxial tension and the simulated local stresses in a ring-shaped hydrogel. (a) The RVE is under uniaxial tension along "1" direction with $\sigma_2 = \sigma_3 = 0$ in the theory. (b-e) Simulated deformation and local stresses of a ring-shape hydrogel upon the same loading as that in experiments with ABAQUS. In the simulation, outer diameter of the ring is 20 mm, the inner diameter of the ring is 16 mm, and the cross-section of the ring is circular. The material is chosen to be neo-hookean with an initial Young's modulus of 130kPa. Due to symmetry, only a quarter of the ring is investigated. The deformed shape and the contour maps of local stresses are provided: (b) Initial shape and deformed shape of a quarter of a soft ring, (c) Contour map of σ_1, (d) Contour map of σ_2, and (e) Contour map of σ_3.

Supplementary Table 1 Kinetic parameters for the mechanical unfolding of CR and LBM*

Protein	Unfolding rate (s^{-1})	Unfolding distance (nm)	Refolding rate (s^{-1})	Refolding distance (nm)	Folded length (nm)	Unfolded length (nm)
GB1	0.039	0.17	720	2.1	2.6	20.6
HP67	2.0×10^{3}	4.8	2.5×10^{5}	5.8	1.8	24.5
SH3	1.0×10^{-5}	2.2	56.7	5.25	0.95	19.6
TIP-1:Kir	0.98 (pulling from N terminus)	0.52				
	0.56 (pulling from C terminus)	0.46				
Coh:Xmod-Doc	7.3×10^{-7} (~85%)	0.13				
	4.7×10^{-4} (~15%)	0.19				

* The unfolding and folding kinetics for GB1, unfolding and folding kinetics for HP67, folding kinetics for SH3, and unfolding kinetics for Coh:Xmod-Doc were taken from Ref. 1, 2, 3, and 4, respectively. [1-4].

Supplementary Table 2 Mechanical properties of ring-shaped Gel-1 after different gelation time in the mold

Gelation time	Number of samples	Failure strain (%)	Failure stress (kPa)	Failure modulus (kPa)	Young's modulus at 5% (kPa)	Young's modulus at 10% (kPa)
2 min	6	16.72±2.11	17.72±3.29	112.67±19.58	152.31±21.13	133.46 ±27.48
1 d	6	16.88±1.96 ^{ns}	17.54±3.03 ^{ns}	107.23±15.49 ^{ns}	157.22±18.76 ^{ns}	127.91 ±21.17 ^{ns}
7 d	6	16.57±2.03 $^{ns, NS}$	17.84±2.91 $^{ns, NS}$	110.37±17.18 $^{ns, NS}$	149.05±20.44 $^{ns, NS}$	128.16 ±19.35 $^{ns, NS}$

The hydrogel was stretched until break (failure) at a constant strain rate of 20 mm min^{-1}. The local slope at a given strain on the stress-strain curve was taken as the Young's modulus at this strain. Student's t-test was used for statistical analyses. When P value is below 0.05, it is considered being statistically significant. Compared with 2 min group: **ns**, not significant; Compared with 1 d group: **NS**, not significant.

Supplementary Table 3 Mechanical properties of ring-shaped Gel-2 after different gelation time in the mold

Gelation time	Number of samples	Failure strain (%)	Failure stress (kPa)	Failure modulus (kPa)	Young's modulus at 50% (kPa)	Young's modulus at 100% (kPa)	Young's modulus at 150% (kPa)	Young's modulus at 200% (kPa)
2 min	6	247.63±27.15	21.68±2.39	8.51±0.93	15.44±1.51	11.60±1.18	10.32±1.05	9.27±0.96
1 d	6	245.26±22.17 ns	22.11±2.23 ns	8.79±0.81 ns	15.18±1.41 ns	11.71±1.11 ns	10.12±1.18 ns	9.17±1.03 ns
7 d	6	249.12±21.39 ns, NS	20.49±2.56 ns, NS	8.21±0.85 ns, NS	15.36±1.47 ns, NS	11.38±1.03 ns, NS	10.46±1.14 ns, NS	9.29±0.92 ns, NS

The hydrogel was stretched until break (failure) at a constant strain rate of 20 mm min^{-1}. The local slope at a given strain on the stress-strain curve was taken as the Young's modulus at this strain. Student's t-test was used for statistical analyses. When P value is below 0.05, it is considered being statistically significant. Compared with 2 min group: **ns**, not significant; Compared with 1 d group: **NS**, not significant.

Supplementary Table 4 Mechanical properties of ring-shaped Gel-3 after different gelation time in the mold

Gelation time	Number of samples	Failure strain (%)	Failure stress (kPa)	Failure modulus (kPa)	Young's modulus at 50% (kPa)	Young's modulus at 100% (kPa)	Young's modulus at 150% (kPa)
2 min	3	212.18±18.89	38.03±3.21	17.69±1.22	27.04±1.08	22.11±1.17	19.36±1.20
1 d	9	211.23±22.17 ns	38.45±3.11 ns	17.98±1.12 ns	27.74±1.36 ns	22.09±1.31 ns	19.44±1.27 ns
7 d	3	209.47±21.36 ns, NS	38.25±3.16 ns, NS	18.23±1.07 ns, NS	27.63±1.21 ns, NS	22.32±1.23 ns, NS	19.47±1.15 ns, NS

The hydrogel was stretched until break (failure) at a constant strain rate of 20 mm min^{-1}. The local slope at a given strain on the stress-strain curve was taken as the Young's modulus at this strain. Student's t-test was used for statistical analyses. When P value is below 0.05, it is considered being statistically significant. Compared with 2 min group: **ns**, not significant; Compared with 1 d group: **NS**, not significant.

Supplementary Table 5 Mechanical properties of ring-shaped Gel-1 at different concentrations

Protein concentration (mg mL^{-1})	Number of samples	Failure strain (%)	Failure stress (kPa)	Failure modulus (kPa)	Young's modulus at 5% (kPa)	Young's modulus at 10% (kPa)
150	9	16.42±1.81	15.08±2.37	98.75±12.34	149.63±16.31	121.71 ±19.54
180	9	16.88±1.96 ns	17.54±3.03 ns	107.23±15.49 ns	157.22±18.76 ns	127.91 ±21.17 ns

The hydrogel was stretched until break (failure) at a constant strain rate of 20 mm min^{-1}. The local slope at a given strain on the stress-strain curve was taken as the Young's modulus at this strain. Student's t-test was used for statistical analyses. When P value is below 0.05, it is considered being statistically significant. **ns**, not significant.

Supplementary Table 6 Mechanical properties of ring-shaped Gel-2 at different concentrations

Protein concentration (mg mL^{-1})	Number of samples	Failure strain (%)	Failure stress (kPa)	Failure modulus (kPa)	Young's modulus at 50% (kPa)	Young's modulus at 100% (kPa)	Young's modulus at 150% (kPa)	Young's modulus at 200% (kPa)
150	9	243.35±26.18	19.05±2.03	7.83±0.61	13.93±1.31	10.89±1.07	9.41±0.86	8.38±0.81
180	9	245.26±22.17 ns	22.11±2.23 **	8.79±0.81 *	15.18±1.41 ns	11.71±1.11 ns	10.12±1.18 ns	9.17±1.03 ns

The hydrogel was stretched until break (failure) at a constant strain rate of 20 mm min^{-1}. The local slope at a given strain on the stress-strain curve was taken as the Young's modulus at this strain. Student's t-test was used for statistical analyses. When P value is below 0.05, it is considered being statistically significant. **ns**, not significant, *$P < 0.05$, **$P < 0.01$.

Supplementary Table 7 Mechanical properties of ring-shaped Gel-3 at different concentrations

Protein concentration (mg mL^{-1})	Number of samples	Failure strain (%)	Failure stress (kPa)	Failure modulus (kPa)	Young's modulus at 50% (kPa)	Young's modulus at 100% (kPa)	Young's modulus at 150% (kPa)
150	9	207.49±21.59	32.02±2.74	15.42±0.82	26.83±1.59	18.51±1.17	16.38±1.12
180	9	211.23±22.17 ns	38.45±3.11 ***	17.98±1.12 ***	22.74±1.36 ***	22.09±1.31 ***	19.44±1.27***

The hydrogel was stretched until break (failure) at a constant strain rate of 20 mm min^{-1}. The local slope at a given strain on the stress-strain curve was taken as the Young's modulus at this strain. Student's t-test was used for statistical analyses. When P value is below 0.05, it is considered being statistically significant. **ns**, not significant, ***$P < 0.001$.

Supplementary Table 8 Mechanical properties of ring-shaped Gel-4 after different gelation time in the mold

Gelation time	Number of samples	Failure strain (%)	Failure stress (kPa)	Failure modulus (kPa)	Young's modulus at 50% (kPa)	Young's modulus at 100% (kPa)	Young's modulus at 150% (kPa)
2 min	3	203.34±20.15	106.07±10.83	53.18±5.68	84.12±8.42	65.12±6.85	58.13±6.89
1 d	9	203.41±19.72 ns	108.26±9.64 ns	53.92±5.31 ns	85.56±7.93 ns	65.87±6.72 ns	58.62±6.61 ns
7 d	3	203.78±19.14 ns, NS	108.79±9.22 ns, NS	53.38±5.02 ns, NS	86.33±7.12 ns, NS	66.11±6.34 ns, NS	58.79±5.82 ns, NS

The hydrogel was stretched until break (failure) at a constant strain rate of 20 mm min^{-1}. The local slope at a given strain on the stress-strain curve was taken as the Young's modulus at this strain. Student's t-test was used for statistical analyses. When P value is below 0.05, it is considered being statistically significant. Compared with 2 min group: **ns**, not significant; Compared with 1 d group: **NS**, not significant.

Supplementary Table 9 Mechanical properties of ring-shaped Gel-4 at different concentrations

**Protein Concentration (mg mL^{-1})**	**Number of samples**	**Failure strain (%)**	**Failure stress (kPa)**	**Breaking modulus (kPa)**	**Young's modulus at 50% (kPa)**	**Young's modulus at 100% (kPa)**	**Young's modulus at 150% (kPa)**
150	9	197.26±17.33	95.15±9.37	48.21±4.12	72.24±7.13	55.93±6.12	49.79±4.47
180	9	203.41±19.72 ns	108.26±9.64 **	53.92±5.31 *	85.56±7.93 **	65.87±6.72 **	58.62±6.61 **

The hydrogel was stretched until break (failure) at a constant strain rate of 20 mm min^{-1}. The local slope at a given strain on the stress-strain curve was taken as the Young's modulus at this strain. Student's t-test was used for statistical analyses. When P value is below 0.05, it is considered being statistically significant. **ns**, not significant, *$P < 0.05$, **$P < 0.01$.

Supplementary Table 10 Parameters for Gel-1 in the simulation

Item	Parameter	Item	Parameter
L_c^f	20.8 nm	N_1	8
ξ	0.4 nm	ΔL_1	18 nm
Ω	3×10^{-29} m^3	k_{10}^f	720 s^{-1}
L_0^{ch}	7 nm	Δz_1^f	2.1 nm
L_0^{cl}	3 nm	k_{10}^{uf}	0.039 s^{-1}
k^{cl}	100 pN nm^{-1}	Δz_1^{uf}	0.17 nm
N	0.0105 nm^{-3}	χ	0.2
γ	0.01 s^{-1}	μ	0^6

Supplementary Table 11 Parameters for Gel-2 in the simulation

Item	Parameter	Item	Parameter	Item	Parameter
L_c^f	17.6 nm	N_1	4	N_2	4
ξ	0.4 nm	ΔL_1	18 nm	ΔL_2	22.7 nm
Ω	3×10^{-29} m^3	k_{10}^f	720 s^{-1}	k_{20}^f	2.5×10^{-5} s^{-1}
L_0^{ch}	6 nm	Δz_1^f	2.1 nm	Δz_2^f	5.8 nm
L_0^{cl}	3 nm	k_{10}^{uf}	0.039 s^{-1}	k_{20}^{uf}	2.0×10^3 s^{-1}
k^{cl}	100 pN nm^{-1}	Δz_1^{uf}	$0.17nm$	Δz_2^{uf}	4.8 nm
N	0.0075 nm^{-3}	χ	0.2	γ	0.01 s^{-1}
μ	0^6				

Supplementary Table 12 Parameters for Gel-3 in the simulation

Item	Parameter	Item	Parameter
L_c^f	7.6 nm	N_1	8
ξ	0.4 nm	ΔL_1	18.65 nm
Ω	3×10^{-29} m^3	k_{10}^f	56.7 s^{-1}
L_0^{ch}	2.7 nm	Δz_1^f	5.25 nm
L_0^{cl}	3 nm	k_{10}^{uf}	0.00001 s^{-1}
k^{cl}	100 pN nm^{-1}	Δz_1^{uf}	2.2 nm
N	0.0103 nm^{-3}	χ	0.2
γ	0.01 s^{-1}	μ	0^6

Supplementary Table 13 Comparison of some parameters used in the simulation for three different gels, as also listed in Supplementary Tables 10-12

	Gel-1	Gel-2	Gel-3
N_1	8	4	8
N_2	0	4	0
L_c^f	20.8 nm	17.6 nm	7.6 nm
N	0.0105 nm^{-3}	0.0075 nm^{-3}	0.0103 nm^{-3}
L_0^{ch}	7 nm	6 nm	2.7 nm

Supplementary Table 14 Comparison of Young's modulus between theory and experiments

	Gel-1	Gel-2	Gel-3
Theory	130 kPa	15 kPa	23 kPa
Experiments	152 kPa	15 kPa	27 kPa

Supplementary Table 15 Comparison of failure strain between theory and experiments

	Gel-1	Gel-2	Gel-3
Theory	17%	150%	210%
Experiments	17%	247%	212%

Supplementary Table 16 Comparison of toughness between theory and experiments

	Gel-1	Gel-2	Gel-3
Theory	4.43×10^{-3} N m^{-1}	17.6×10^{-3} N m^{-1}	46.2×10^{-3} N m^{-1}
Experiments	3.78×10^{-3} N m^{-1}	48×10^{-3} N m^{-1}	83.9×10^{-3} N m^{-1}

SUPPLEMENTARY METHODS

Reagents

O-Benzotriazole-N,N,N',N'-tetramethyl-uronium-hexafluorophosphate (HBTU) was obtained from NovaBioChem. Aminopropyltriethoxysilane (APTES), Tris (2-carboxyethyl) phosphine hydrochloride (TCEP), Diisopropylethyl amine (DIPEA) and piperidine were purchased from Sigma-Aldrich. Maleimide-PEG-NHS (MW: 5000) and Methyl-PEG-NHS (MW: 2000) were from Laysan Bio, Inc. (AL, USA). Chlorotrityl chloride resin, Fmoc-Ile-OH, Fmoc-Ala-OH, Fmoc-Ser(tBu)-OH, Fmoc-Glu(OtBu)-OH, Fmoc-Arg(Pbf)-OH, Fmoc-Trp(Boc)-OH, Fmoc-Tyr(tBu)-OH, Fmoc-Ser(tBu)-OH, Fmoc-Ile-OH, Fmoc-Asn(Tyt)-OH, Fmoc-Cys(Trt)-OH Fmoc-Gly-OH, Fmoc-Ala-OH and Fmoc-Tyr(tBu)−OH were received from GL Biochem (Shanghai, China). All other chemicals were obtained from Aladdin. All solvents were used directly without further purification.

Synthesis of Kir peptide

The Kir peptide with the sequence of CNISYWRESAI was synthesized via the common solid phase peptide synthesis protocol. First, Fmoc-Ile-OH (1 equiv.) and DIPEA (4 equiv.) were dissolved in CH_2Cl_2 (20 mL g^{-1} resin). Then chlorotrityl chloride resin (1 equiv.) was added to the solution and the mixture was stirred at room temperature for 1 h. The reaction mixture was filtered and the unreacted resin was capped with 1: 2: 17 (v/v/v) DIPEA-MeOH-DCM (3 × 20 mL per gram resin). After the capping procedure, the resin was thoroughly rinsed with CH_2Cl_2, DMF, and CH_2Cl_2, and then dried in vacuo. The bead-loading was determined to be ~ 0.5 mmol g^{-1} by the 2% DBU/DMF method. The beads were then swollen in DMF for 0.5 h in a sealed spin column and DMF was then removed via filtration. Then 20% Piperidine/DMF (3 × 5 mL, 5 min

each time) was added to remove the Fmoc protecting group. The sequence was elongated with the HBTU coupling reaction: Fmoc-Ala-OH, Fmoc-Ser(tBu)-OH, Fmoc-Glu(OtBu)-OH, Fmoc-Arg(Pbf)-OH, Fmoc-Trp(Boc)-OH, Fmoc-Tyr(tBu)-OH, Fmoc-Ser(tBu)-OH, Fmoc-Ile-OH, Fmoc-Asn(Tyt)-OH, or Fmoc-Cys(Trt)-OH (4 equiv.), HBTU (4 equiv.), and DIPEA (8 equiv.) were dissolved in DMF (~ 5 mL) and then transferred to the de-Fmoc resin. The mixture was shaken at room temperature for 2 hr for elongation of the sequence. Finally, the resin was washed thoroughly with DMF, CH_2Cl_2, and DMF. Reagent K (TFA/thioanisole/water/phenol/EDT = 82.5:5:5:5:2.5, V/V; 75 mL per gram of resin) was incubated with the resin for ~ 3 h at room temperature to cleave the peptide from the resin and to remove the protecting groups on the side chains of the amino acids in the same step. The mixture was filtered and the solution was concentrated in vacuo. Then the residue was precipitated with cold diethyl ether. The product was collected by centrifuge, washed with diethyl ether, and then dried under high vacuum. The obtained CNISYWRESAI was characterized by HPLC (Supplementary Figure 9) and mass spectroscopy (Supplementary Figure 10), and the purity is more than 96%. HPLC conditions: Alltima™ C18 4.6 x 250 mm column, solvent A: 0.065% TFA in H_2O (v/v), solvent B: 0.05% TFA in CH_3CN (v/v), gradient: 0 to 2.6 min, 1% A, 2.6 to 7.65 min, 1% to 5% A, 7.65 to 17.8 min, 5% to 99% A, 17.8 to 20.15 min, 99% to 100% A, 20.15 to 24 min, 100% to 99% A, 24 to 34 min, 99% A.

Protein expression and purification

The gene encoding polyproteins TIP-1-$(GB1)_8$-TIP-1, TIP-1-$(GB1\text{-}Hp67)_4$-TIP-1, TIP-1-$(SH3)_8$-TIP-1, Coh-$(GB1)_4$-cys and cys-Xmod-Doc were constructed using standard molecular biology techniques based on published procedures with slight modification [1,7-9]. The gene for GB1 was a

gift from Prof. Hongbin Li. The gene for HP67 was a gift from Prof. Robert Robinson. The genes for SH3, TIP-1, Coh and Xmod-Doc were purchased from GenScript, Inc. (Nanjing, China). The gene for Snap tag was purchased from NEB. All genes for the protein modules were flanked with 5' BamHI and 3' BglII and KpnI restriction sites. The engineering of the polyprotein genes was done in a stepwise fashion based on the same sticky ends generated by BamHI and BglII. For example, to generate TIP-1-$(GB1)_8$-TIP-1, the plasmid for TIP-1 gene was digested with BamHI and KpnI, resulting in an TIP-1 insert with overhanging "sticky ends" whose sequence corresponded to that of the pQE80L vector digested with the same enzymes. The sticky-ended TIP-1 insert was subsequently ligated into the digested pQE80L vector to form pQE80L-TIP-1. In a similar way, Digestion of $(GB1)_8$ with BamHI and KpnI resulted in overhanging "sticky ends" whose sequence corresponded to that of the pQE80L-Tip1 vector digested with BglII and KpnI. The sticky-ended $(GB1)_8$ insert was subsequently ligated into the digested pQE80L-TIP-1 vector to form pQE80L-TIP-1-$(GB1)_8$. Sticky ended TIP-1 gene was subsequently cloned into the pQE80L-TIP-1-$(GB1)_8$ vector to form pQE80L-TIP-1-$(GB1)_8$-TIP-1 in a similar manner. The pQE80L-TIP-1-$(GB1\text{-}Hp67)_4$-TIP-1 and pQE80L-TIP-1-$(SH3)_8$-TIP-1 vectors were constructed in a similar way. The pQE80L vectors containing Coh-$(GB1)_4$-cys or cys-Xmod-Doc were also constructed in a similar way. The correctness of the sequence for the polyproteins were confirmed by direct sequencing. The protein sequences are listed below.

TIP-1-$(GB1)_8$-TIP-1:

MRGSHHHHHHGSMSYIPGQPVTAVVQRVEIHKLRQGENLILGFSIGGGIDQDPSQNPFSE
DKTDKGIYVTRVSEGGPAEIAGLQIGDKIMQVNGWDMTMVTHDQARKRLTKRSEEVV
RLLVTRQSLQKAVQQSMLSRSMDTYKLILNGKTLKGETTTEAVDAATAEKVFKQYAND
NGVDGEWTYDDATKTFTVTERSMDTYKLILNGKTLKGETTTEAVDAATAEKVFKQYA

NDNGVDGEWTYDDATKTFTVTERSMDTYKLILNGKTLKGETTTEAVDAATAEKVFKQ
YANDNGVDGEWTYDDATKTFTVTERSMDTYKLILNGKTLKGETTTEAVDAATAEKVF
KQYANDNGVDGEWTYDDATKTFTVTERSMDTYKLILNGKTLKGETTTEAVDAATAEK
VFKQYANDNGVDGEWTYDDATKTFTVTERSMDTYKLILNGKTLKGETTTEAVDAATA
EKVFKQYANDNGVDGEWTYDDATKTFTVTERSMDTYKLILNGKTLKGETTTEAVDAA
TAEKVFKQYANDNGVDGEWTYDDATKTFTVTERSMDTYKLILNGKTLKGETTTEAVD
AATAEKVFKQYANDNGVDGEWTYDDATKTFTVTERSMSYIPGQPVTAVVQRVEIHKLR
QGENLILGFSIGGGIDQDPSQNPFSEDKTDKGIYVTRVSEGGPAEIAGLQIGDKIMQVNG
WDMTMVTHDQARKRLTKRSEEVVRLLVTRQSLQKAVQQSMLSRS

TIP-1-(GB1-Hp67)$_4$-TIP-1:

MRGSHHHHHHGSMSYIPGQPVTAVVQRVEIHKLRQGENLILGFSIGGGIDQDPSQNPFSE
DKTDKGIYVTRVSEGGPAEIAGLQIGDKIMQVNGWDMTMVTHDQARKRLTKRSEEVV
RLLVTRQSLQKAVQQSMLSRSMDTYKLILNGKTLKGETTTEAVDAATAEKVFKQYAND
NGVDGEWTYDDATKTFTVTERSMSGPLPIFPLEQLVNKPVEELPEGVDPSRKEEHLSIED
FTQAFGMTPAAFSALPRWKQQNLKKEKGLFRSMDTYKLILNGKTLKGETTTEAVDAAT
AEKVFKQYANDNGVDGEWTYDDATKTFTVTERSMSGPLPIFPLEQLVNKPVEELPEGV
DPSRKEEHLSIEDFTQAFGMTPAAFSALPRWKQQNLKKEKGLFRSMDTYKLILNGKTLK
GETTTEAVDAATAEKVFKQYANDNGVDGEWTYDDATKTFTVTERSMSGPLPIFPLEQL
VNKPVEELPEGVDPSRKEEHLSIEDFTQAFGMTPAAFSALPRWKQQNLKKEKGLFRSMD
TYKLILNGKTLKGETTTEAVDAATAEKVFKQYANDNGVDGEWTYDDATKTFTVTERS
MSGPLPIFPLEQLVNKPVEELPEGVDPSRKEEHLSIEDFTQAFGMTPAAFSALPRWKQQN
LKKEKGLFRSMSYIPGQPVTAVVQRVEIHKLRQGENLILGFSIGGGIDQDPSQNPFSEDKT

DKGIYVTRVSEGGPAEIAGLQIGDKIMQVNGWDMTMVTHDQARKRLTKRSEEVVRLLV

TRQSLQKAVQQSMLSRS

TIP-1-(SH3)$_8$-TIP-1:

MRGSHHHHHHGSMSYIPGQPVTAVVQRVEIHKLRQGENLILGFSIGGGIDQDPSQNPFSE

DKTDKGIYVTRVSEGGPAEIAGLQIGDKIMQVNGWDMTMVTHDQARKRLTKRSEEVV

RLLVTRQSLQKAVQQSMLSRSTFVALYDYESRTETDLSFKKGERLQIVNNTEGDWWLA

HSLTTGRTGYIPSNYVAPSDRSTFVALYDYESRTETDLSFKKGERLQIVNNTEGDWWLA

HSLTTGRTGYIPSNYVAPSDRSTFVALYDYESRTETDLSFKKGERLQIVNNTEGDWWLA

HSLTTGRTGYIPSNYVAPSDRSTFVALYDYESRTETDLSFKKGERLQIVNNTEGDWWLA

HSLTTGRTGYIPSNYVAPSDRSTFVALYDYESRTETDLSFKKGERLQIVNNTEGDWWLA

HSLTTGRTGYIPSNYVAPSDRSTFVALYDYESRTETDLSFKKGERLQIVNNTEGDWWLA

HSLTTGRTGYIPSNYVAPSDRSTFVALYDYESRTETDLSFKKGERLQIVNNTEGDWWLA

HSLTTGRTGYIPSNYVAPSDRSTFVALYDYESRTETDLSFKKGERLQIVNNTEGDWWLA

HSLTTGRTGYIPSNYVAPSDRSMSYIPGQPVTAVVQRVEIHKLRQGENLILGFSIGGGIDQ

DPSQNPFSEDKTDKGIYVTRVSEGGPAEIAGLQIGDKIMQVNGWDMTMVTHDQARKRL

TKRSEEVVRLLVTRQSLQKAVQQSMLSRS

Coh-(GB1)$_4$-cys:

MRGSHHHHHHGSMGTALTDRGMTYDLDPKDGSSAATKPVLEVTKKVFDTAADAAGQ

TVTVEFKVSGAEGKYATTGYHIYWDERLEVVATKTGAYAKKGAALEDSSLAKAENNG

NGVFVASGADDDFGADGVMWTVELKVPADAKAGDVYPIDVAYQWDPSKGDLFTDNK

DSAQGKLMQAYFFTQGIKSSSNPSTDEYLVKANATYADGYIAIKAGEPRSMDTYKLILN

GKTLKGETTTEAVDAATAEKVFKQYANDNGVDGEWTYDDATKTFTVTERSMDTYKLI

LNGKTLKGETTTEAVDAATAEKVFKQYANDNGVDGEWTYDDATKTFTVTERSMDTYK

LILNGKTLKGETTTEAVDAATAEKVFKQYANDNGVDGEWTYDDATKTFTVTERSMDT

YKLILNGKTLKGETTTEAVDAATAEKVFKQYANDNGVDGEWTYDDATKTFTVTERSC

cys-Xmod-Doc:

MRGSHHHHHHGSCGGNTVTSAVKTQYVEIESVDGFYFNTEDKFDTAQIKKAVLHTVYN

EGYTGDDGVAVVLREYESEPVDITAELTFGDATPANTYKAVENKFDYEIPVYYNNATLK

DAEGNDATVTVYIGLKGDTDLNNIVDGRDATATLTYYAATSTDGKDATTVALSPSTLV

GGNPESVYDDFSAFLSDVKVDAGKELTRFAKKAERLIDGRDASSILTFYTKSSVDQYKD

MAANEPNKLWDIVTGDARS

The primer sequences to generate each building blocks with the N-terminal *BamH*I (GGATCC) site and C-terminal *Bgl*II (AGATCT)and *Kpn*I (GGTACC) sites are listed below.

TIP-1:

TIP-1_up: 5'-CTCGGATCCATGTCATACATTCCGGGC-3'

TIP-1_down: 5'-GCTGGTACCCTATTAAGATCTAGACAGCATGCTTTGC-3'

GB1:

GB1_up: 5'-CTCGGATCCATGGACACCTACAAAC-3'

GB1_down: 5'-GCTGGTACCCTATTAAGATCTTTCGGTAACCGTG-3'

GB1-cys_down: 5'-GCTGGTACCCTATTAACAAGATCTTTCGGTAACCGTG-3'

HP67:

HP67_up: 5'-CGCGGATCCATGTCTGGGCCTCTGCCCATCTTC-3'

HP67_down: 5'-CGGGGTACCTAATAAAGATCTAAATAGTCCTTTTCTTTCTTGAGG-3'

SH3:

SH3_up: 5'-CTCGGATCCGAAAATCTGTACTTC-3'

SH3_down: 5'-GCTGGTACCCTATTAAGATCTGCTCGGTGCGACGTAG -3'

Coh:

Coh_up: 5'-CTCGGATCCATGGGCACCGC-3'

cys-Xmod-Doc: 5'-GCTGGTACCCTATTAAGATCTCGGTTCACCCG-3'

cys-Xmod-Doc:

cys-Xmod-Doc_up: 5'-CTCGGATCCTGCGGCGGTAACAC-3'

cys-Xmod-Doc_down: 5'-GCTGGTACCCTATTAAGATCTGGCATCGCCGGTAAC-3'

The plasmid was then transformed into the *Escherichia coli* strain BL21. Cultures were grown in 2.5% LB containing 100mg/L ampicillin at 37 °C, and protein expression was induced with 1 mM isopropyl-1-β-D-thiogalactoside (IPTG) when the optical density at 600 nm reached ~0.7. Protein expression continued for 6 hours. The cells were harvested by centrifugation at 6,000 g for 15 min and the cell lysis was done by ultrasound using a probe sonicator. The soluble protein was purified by Co^{2+} affinity column. The yield of TIP-1-$(GB1)_8$-TIP-1, TIP-1-$(GB1\text{-}Hp67)_4$-TIP-1 ,TIP-1-$(SH3)_8$-TIP-1, Coh-$(GB1)_4$-cys and cys-Xmod-Doc was 10–30 mg per liter of bacteria culture. The purified protein was dialyzed against deionized water for two days to remove all the salts (Dialysis membranes MWCO 3, 000; Pierce Co., Wisconsin, USA), and subsequently lyophilized. The purity of TIP-1-$(GB1)_8$- TIP-1, TIP-1-$(GB1\text{-}Hp67)_4$- TIP-1 and TIP-1-$(SH3)_8$- TIP-1 was confirmed by SDS-PAGE to be more than 90% (Supplementary Figure 11).

AFM tip/Substrate surface modification

The cantilevers for the measurements of the mechanical properties of LBMs were used directly without any modification.

For the measurement of the binding forces for the TIP-1:Kir complex, the cantilever modification procedure was similar as that reported in our previous study[10-13]. Briefly, the MLCT cantilever from Bruker was treated with hot chromic acid solution to remove the organic impurities and generate hydroxyl groups for 20 min. After extensive rinsing with deionized water, the cantilever was dried and then placed in a toluene solution containing 0.5% (vol/vol) 3-aminopropyl triethoxysilane (APTES) for 2 h to introduce amino groups to the cantilever tip surface. Afterward, the cantilever was rinsed with toluene, dried under a nitrogen stream, and further incubated in a drying oven at 80°C for 20 min. Next the cantilever was immersed in a dimethyl sulfoxide (DMSO) solution containing 1mg mL^{-1} of maleimide-polyethylene glycol-N-hydroxysuccinimide (MAL-PEG-NHS) (MW: 5000 Da, Nanocs) for 3 h to link the PEG linker to the surface via NH_2-NHS reaction. Finally, the cantilever was rinsed with deionized water and placed in a solution containing Kir peptide to conjugate the peptide to the PEG linker via maleimide-thiol reaction. The cantilever was immediately used for single molecule force spectroscopy experiments after modification.

For the measurement of the binding forces for the Xmod-Doc:Coh complex, the MLCT cantilever from Bruker was used. The cantilevers were treated with hot chromic acid solution to remove the organic impurities and generate hydroxyl groups for 20 minutes. After extensive rinsing with deionized water, the cantilever was dried and then placed in a DMSO solution containing silane-PEG-Mal (MW: 5000 Da, 1 mg mL^{-1}, Nanocs) for 2 h. The cantilever was immersed into cys-Xmod-doc solution (0.1 mg mL^{-1}) for 2 h to allow proteins bind to the cantilever tip via gold-thiol bond. Then, the cantilever was washed and stored in TBS buffer with calcium ions (25 mM Tris, 72 mM NaCl, 1 mM $CaCl_2$, pH = 7.4).

Glass substrates were first immersed into chromic acid for 2 h to remove impurities. After rinsing with deionized water, the glass slides were dried by blowing with nitrogen gas and then placed in a DMSO solution containing silane-PEG-NHS (MW: 5000 Da, 1 mg mL^{-1}, Nanocs) for 2 h. The glass slides were extensively rinsed with DMSO to remove the unreacted silane-PEG-NHS. Then they were covered by NH_2-BG solution in DMSO (10 μg mL^{-1}) for 2 h so that Snap protein can directly bind to the surface via BG-Snap covalent bond. Finally, Subsequently, the surface was rinsed with deionized water to remove unreacted NH_2-BG. The glass substrates were used immediately after modification.

Single molecule force spectroscopy experiments and data analysis

Single-molecule AFM experiments were carried out on a commercial AFM (ForceRobot 300, JPK, Berlin, Germany) as described previously [10-13]. All the force-extension experiments for the polyproteins were carried out in PBS buffer (10 mM phosphate, 137 mM NaCl, pH 7.4) at room temperature (~22 °C). Protein samples (0.1 mg mL^{-1},150 μL) was directly deposited on the freshly prepared glass surface for 2 h and then washed with buffer to remove unadsorbed proteins. Then, the sample chamber was filled with 1mL buffer before the measurement. The spring constants of the AFM cantilevers (Biolever-RC-150VB-70 from Olympus or MLCT from Bruker) were calibrated using the equipartition theorem before each experiment, with typical values of 6 and 50 pN nm^{-1}, respectively. The pulling speed was 400 nm s^{-1} for all traces unless otherwise specified.

The single molecule force spectroscopy for the Tip-1:Kir complex were conducted in PBS buffer (10 mM phosphate, 137 mM NaCl, pH 7.4) at room temperature (~22 °C).

The single molecule force spectroscopy for Xmod-Doc:Coh complex were conducted in TBS buffer (25 mM Tris, 72 mM NaCl, 1 mM $CaCl_2$, pH=7.4) at room temperature (~22 °C).

Preparation of Kir modified 4-Armed PEG multivalent crosslinker (MCL)

The Kir peptide (sequence: CNISYWRESAI), a TIP-1 recognizing and binding peptide, comprises of a 11 amino-acid sequence derived from ten C-terminal residues of the PDZ-recognition peptide [14,15] and an N-terminal cysteine for conjugation with the maleimide group at the end of each arm of the 4-armed PEG through thiol-ene reaction. Maleimide-end-capped four-armed poly(ethylene glycol) with the molecular weight of 10 kDa was purchased from Laysan Bio, Inc. (AL, USA). The functionality of maleimide was determined as 4.03 per molecule by ultraviolet (UV) measurement at 297 nm. The conjugation of 4-Armed PEG with the kir peptide was conducted according to the method by Ito et al. with minor modification [16]. A mixture of the kir peptide and maleimidated 4-Armed PEG (the molar ratios of the peptide to maleimide group was 5:1) in dimethyl formamide (DMF)was gently stirred overnight. Subsequently the reaction product was dialyzed against deionized water (Dialysis membranes MWCO 3000; Pierce Co.) for 3 h, repeating for 5 times, to remove DMF and the unreacted peptides. Finally, the samples were lyophilized to give a white powder of the multi-crosslinker of 4-Armed PEG-Kir. The purity was determined to be ~90% based on the HPLC trace. The major impurities were the products that linked with less than 4 Kir peptides per molecule (Supplementary Figure 12a). The conjugation ratio increased with the increase of the molar ratios of the peptide to maleimide group (Supplementary Figure 12b) and in average ~3.9 out of 4 arms of the PEG were successfully linked with Kir peptides as determined by ITC titration (Supplementary Figure 12c).

Preparation of cys-Xmod-Doc terminated 4-Armed PEG MCL

The protein cys-Xmod-Doc was capped with cysteine at its N-terminus, which can react with maleimidated PEG through thiol-ene reaction. Maleimide-end-capped four-armed poly(ethylene glycol) with the molecular weight of 10k Da was purchased from Laysan Bio, Inc. (AL, USA). The functionality of maleimide was determined as 4.03 per molecule by ultraviolet (UV) measurement at 297 nm. The conjugation of 4-Armed PEG with the protein cys-Xmod-Doc was conducted according to the method by Ito et al. with minor modification[16]. A mixture of cysteine-end-capped protein cys-Xmod-Doc, maleimidated 4-Armed PEG (the molar ratios of the protein to maleimide group was 5:1) and TCEP (tris(2-carboxyethyl)phosphine, 5 mM) in PBS (pH7.0) was gently stirred at room temperature overnight. Subsequently, the reaction product was dialyzed against deionized H_2O (Dialysis membranes MWCO 100, 000; Pierce Co.) for 1 h, repeating for 5 times, to remove salt, unreacted proteins and PEG. Finally, the sample was lyophilized to give a white powder of cys-Xmod-Doc conjugated 4-Armed PEG (Named as 4-Armed PEG-Xmod-Doc). The purity was confirmed by SDS-PAGE to be ~90%.

Preparation of Coh-$(GB1)_4$-SS-$(GB1)_4$-Coh

After the protein Coh-$(GB1)_4$-cys has been purified by Co^{2+} affinity column, the purified protein was dialyzed against PBS (pH7.4) for 3 d to get Coh-$(GB1)_4$-cys-cys-$(GB1)_4$-Coh by the formation of the disulfide bond between Coh-$(GB1)_4$-cys during dialysis. The dimerization of the protein was very efficient without the need of additional oxidants, as the cys residue after GB1 is highly reactive. Then the resulted product was dialyzed against deionized water (Dialysis membranes MWCO 3, 000; Pierce Co.) for 2 h, repeating for 5 times, to remove salts in the PBS

buffer. Finally, the sample was lyophilized to give a white powder of Coh-$(GB1)_4$-SS-$(GB1)_4$-Coh. The purity was higher than 90% as confirmed by SDS-PAGE.

Hydrogel Preparation

Preparation of the hydrogel was based on the specific protein-peptide interaction between TIP-1 and the Kir peptide or the specific protein-protein interaction between Xmod-Doc and Coh. The lyophilized ABA proteins and MCL were re-dissolved in phosphate saline buffer (PBS, pH7.4), respectively. Then the two solutions were successively added into a custom-made silicone rubber mold with a ring-shaped slot (d_{in} = 15 mm, d_{out} = 21 mm, h = 6 mm). The mold containing the mixture of protein and 4-Armed PEG-Peptide (the final mole ratio of protein to 4-Armed PEG-Peptide = 2:1) was remained stationary for at least 2 minutes and the ring-shaped hydrogel sample was then carefully taken out of the mold and stored in PBS buffer before mechanical test. The gelation was very fast, the mechanical properties of the hydrogel samples after gelation for more than 2 min were similar. All hydrogel samples prepared in this way were quite stable with small swelling ratios, and can be stored for one year without significant erosion. The swelling ratios and erosion profiles are summarized in Supplementary Figure 2 and Supplementary Figure 3, respectively. The hydrogels made of folded protein domains typically do not swell too much in PBS buffer. It might be because they are relatively hard to change their conformations comparing with the unstructured proteins due to the existence of special intra-chain interactions.

Tensile Test

Tensile tests were performed using an Instron-5944 tensometer. The force gauge (load cell) used for all experiments is a 10 N Static Load Cell (Instron, Catalog no. 2530-10N; Force capacity: ±10 N; Linearity: ±0.25% of reading from 0.4 to 100% of force capacity; Repeatability: 0.25% of reading from 0.4 to 100% of force capacity; Temperature Effect on Sensitivity: ±0.002% of force capacity per °C (0.001% per °F)). Unless otherwise noted, these tests were done in PBS buffer (10 mM phosphate, 137 mM NaCl, pH 7.4) at a constant temperature of 20 °C. In a typical experiment, the ring-shaped hydrogel was hooked by a fixed hook on the top and stretched by another hook on the bottom [7]. The hydrogel was pre-equilibrated in the buffer for at least 1 h before mechanical testing. No additional pre-conditioning of the hydrogel sample was performed for stabilization of viscoelastic properties. The hydrogel was then stretched to the given length at a constant strain rate of 20 mm min^{-1}. The local slope at a given strain on the stress-strain curve was taken as the Young's modulus at this strain. For stretching-relaxation experiments, the hydrogels were first gently stretched to flatten the ring and align it to the force direction. This position was set as zero strain. The hydrogels were stretched from this position to a given strain (provided in the figure) and then relaxed back to zero strain or a specific residual strain (provided in the figure) at a strain rate of 20 mm min^{-1}.

The test regimes for all the experiments are list below:

Figure 3

In figure 3c-e, the hydrogel was stretched until break at a constant strain rate of 20 mm min^{-1}.

In figure 3f, the hydrogel was stretched to the given length (5% or 10% strain, respectively), and then immediately relaxed to 0% strain. All the experiments were at a constant strain rate of 20 mm min^{-1}.

In figure 3g-h, the hydrogel was stretched to the given length (10%, 20%, 40%, 60%, 80%, 100%, 120%, 140%, 160% or 180% strain, respectively), and then immediately relaxed to 0% strain. All the experiments were at a constant strain rate of 20 mm min^{-1}.

Figure 5

In figure 5f, the hydrogel (Gel-4) was stretched until break at a constant strain rate of 20 mm min^{-1}.

In figure 5g, the hydrogel (Gel-4) was stretched to the given length (10%, 20%, 40%, 60%, 80%, 100%, 120%, 140%, 160% strain, respectively), and then immediately relaxed to 0% strain. All the experiments were at a constant strain rate of 20 mm min^{-1}.

In figure 5h, the hydrogel (Gel-4) was stretched to the given length (20%, 40%, 60%, 80%, 100%, 120%, 140%, 160% strain, respectively), and then immediately relaxed to 0% strain. There are totally 5 continuous cycles without any waiting time between each cycle. All the experiments were at a constant strain rate of 20 mm min^{-1}.

In figure 5i, the hydrogel (Gel-4) was stretched to 160% strain. Immediately after that, the hydrogel (Gel-4) was relaxed to the given residual strain (0%, 20%, 60%, 100%, 140% strain, respectively). There are totally 5 continuous cycles without any waiting time between each cycle. All the experiments were at a constant strain rate of 20 mm min^{-1}.

In figure 5k, the pristine or the self-healed hydrogel (Gel-4) was stretched until break at a constant strain rate of 20 mm min^{-1}.

In figure 5l, the pristine or the self-healed hydrogel (Gel-4) was stretched to 160% strain, and then immediately relaxed to 0% strain. All the experiments were at a constant strain rate of 20 mm min^{-1}.

Supplementary Figure 4

In Supplementary Figure 4, the hydrogel was stretched until break at a constant strain rate of 20 mm min^{-1}.

Supplementary Figure 5

In Supplementary Figure 5, the hydrogel (Gel-3) was stretched to the given length (20%, 40%, 60%, 100%, 140%, 160%, 180% strain, respectively), and then immediately relaxed to 0% strain. There are totally 5 continuous cycles with indicated waiting time between each cycle. All the experiments were at a constant strain rate of 20 mm min^{-1}.

Supplementary Figure 6

In Supplementary Figure 6, the hydrogel (Gel-2 or Gel-3) was stretched to 180% strain. Immediately after that, the hydrogel was relaxed to the given residual strain following the experimental protocol shown in Supplementary Figure 6a. There are totally 5 continuous cycles without any waiting time between each cycle. All the experiments were at a constant strain rate of 20 mm min^{-1}.

Supplementary Figure 7

In Supplementary Figure 5c-e, the pristine or the self-healed hydrogel was stretched until break at a constant strain rate of 20 mm min^{-1}.

Supplementary Figure 8

In Supplementary Figure 6, the pristine or the self-healed hydrogel was stretched to 140% strain, and then immediately relaxed to 0% strain. All the experiments were at a constant strain rate of 20 mm min^{-1}.

Self-healing Experiment

The broken ring-shaped hydrogel was put back into mold and clamped to bring the two broken interfaces together. PBS buffer was used to cover the ring to avoid dehydration of the ring during the self-healing process. After a preset time, the ring-shaped hydrogel was very carefully take out for further mechanical test. The ring-shaped hydrogel can be partially healed and remain intact when healing time was more than 1 min. The statistics for recovery of the stain and stress of the self-healed hydrogels are summaried in Supplementary Figure 13 and 14, respectively.

Constitutive modeling of hydrogels with folded domains

The synthetic material, schematically shown in Figure 4a, is represented with a volume element of a cube in Fig. 4b. Within this cube, each of 8 protein chains is crosslinked with one arm of linker proteins extending from the cubic center and with that from each corner, essentially similar to the 8-chain model[17]. At the dry state, the cube of RVE is of dimension, l_d. Due to solvent absorption or mechanical loading, the represented volume element (RVE) is deformed and can become rectangular within principal axes of stretches. At the current state, the dimensions of RVE become l_1, l_2, and l_3.

To a good approximation, the volume of the RVE at the current state is equal to the sum of the volume of a dry protein network and that of the absorbed water[6], i.e.,

$$l_1 l_2 l_3 = l_d^3 + \Omega M \qquad (1)$$

where Ω is the volume per water molecule and M is the number of water molecules.

At the current state, the principal stretches of the cube, denoted as λ_1, λ_2, and λ_3, respectively, are given by

$$\lambda_1 = l_1 / l_d$$
$$\lambda_2 = l_2 / l_d$$
$$\lambda_3 = l_3 / l_d \,. \qquad (2)$$

Denote the stretch of one arm of linker proteins as λ^{cl}, that of a protein chain as λ^{ch}. The geometric constraint enforces that

$$\lambda^{ch} L_0^{ch} + \lambda^{cl} L_0^{cl} = \frac{\sqrt{3}}{2} \lambda l_d \,, \qquad (3)$$

where $\lambda = \sqrt{\frac{\lambda_1^2+\lambda_2^2+\lambda_3^2}{3}}$, L_0^{ch} is the reference length of two arms of linker proteins at the dry state, and L_0^{ch} is that of a protein chain at the dry state.

Dividing both sides of Supplementary Eq. 1 by l_d^3 , we have

$$1 + \Omega C = \lambda_1 \lambda_2 \lambda_3 \,, \qquad (4)$$

where $C = M/l_d^3$ is the nominal concentration of water.

When protein chains are subjected to force, F, their originally folded domains can be unfolded. In single molecule force spectroscopy experiments, the variation of the peak force values is an intrinsic feature because unfolding of folded domains within a polyprotein chain is stochastic. The broadness of the force peak distribution is roughly reversely proportional to the width of the potential well underlying the unfolding pathway. Such broad force distribution can be adequately modeled using the Bell's law. Therefore, in the theory, we used the kinetic parameters (spontaneous unfolding rate and unfolding distance) extracted from the unfolding events based on the Bell's law to model unfolding of proteins under force, which can adequately reproduce the

large variation of the peak force values observed in experiments. Consider that each protein chain has two types of folded domains, Type 1 domain and Type 2 domain, that are linked in tandem. The unfolding rate of each type of folded domain, denoted as k_{α}^{uf}, is given by [18]

$$k_{\alpha}^{uf} = k_{\alpha 0}^{uf} \exp\left(\frac{F \Delta z_{\alpha}^{uf}}{k_B T} \right), \qquad (5)$$

where $\alpha = 1$ or Type 1 domain, $\alpha = 2$ for Type 2 domain, $k_{\alpha 0}^{uf}$ is the unfolding rate at $F = 0$, Δz_{α}^{uf} is the distance to the transition state on the unfolding pathway, k_B is the Boltzmann constant, and T is the absolute temperature.

The unfolded domains can be re-folded with a folding rate, denoted as k_{α}^{f}, given by [18,19]

$$k_{\alpha}^{f} = k_{\alpha 0}^{f} \exp\left(-\frac{F \Delta z_{\alpha}^{f}}{k_B T} \right), \qquad (6)$$

where $k_{\alpha 0}^{f}$ is the folding rate at $F = 0$ and Δz_{α}^{f} is the distance to the transition state on the folding pathway.

The fraction of unfolded domains of each type within a protein chain, Λ_{α} ($\alpha = 1, 2$), evolves as

$$\frac{d\Lambda_{\alpha}}{dt} = k_{\alpha}^{uf}(1 - \Lambda_{\alpha}) - k_{\alpha}^{f}\Lambda_{\alpha}. \qquad (7)$$

The contour length of the protein chain at the current state, denoted as L_c, changes due to domains being folded or unfolded and is calculated as

$$L_c = L_c^f + \Lambda_1 N_1 \Delta L_1 + \Lambda_2 N_2 \Delta L_2, \qquad (8)$$

where L_c^f is the contour length of a chain in the fully folded state, N_{α} ($\alpha = 1, 2$)is the total number of folded domains of each type within a protein chain, and ΔL_{α} ($\alpha = 1, 2$) is the change in contour length when a single folded domain of Type 1 or Type 2 is unfolded.

The force-stretch curve of a protein chain is described by the worm-like chain theory, given by [20]

$$F = \frac{k_B T}{\xi}\left[\frac{1}{4}\left(1-\frac{x}{L_c}\right)^{-2} - \frac{1}{4} + \frac{x}{L_c}\right], \qquad (9)$$

where ξ is the persistence length of the protein chain and $x = (\lambda^{ch} - 1)L_0^{ch}$. Two arms of linker proteins in series with a protein chain behaves like a linear elastic spring so that F should also satisfy

$$F = \left(\lambda^{cl} - 1\right)k^{cl}L_0^{cl}, \qquad (10)$$

where k^{cl} is the spring constant.

Bonds formed between protein chains and arms of linker proteins may break and re-associate, which has not been considered in the current analysis for simplicity. The failure stress for Gel-1, Gel-2 and Gel-3 is set to be 20 kPa when determing the failure strain in the analysis.

In the theory, the current state of a block is defined by λ_1, λ_2, λ_3, and Λ_α. It is envisaged that the elastic equilibrium takes place at a much smaller time scale than single stochastic event, including domains being folded or unfolded. This would allow us to decouple the elasticity from the stochastic.

To solve the elastic field, we employ the principle of virtual work. At fixed Λ_α, we let the RVE at the current state change its dimensions by infinitesimal small amounts, δl_1, δl_2, and, δl_3. The resulting virtual work done by external forces, denoted as P_1, P_2, and P_3 respectively, will be $P_1\delta l_1 + P_2\delta l_2 + P_3\delta l_3$. Meanwhile, the number of water molecules in the block would increase by δM and the virtual work done by the chemical potential of water should be $\mu\delta M$. According to the principle of virtual work, the sum of the virtual work done by the applied forces and that by the chemical potential of water should be equal to the change in the internal energy within the RVE [6], denoted as δu, i.e.,

$$\delta u = P_1\delta l_1 + P_2\delta l_2 + P_3\delta l_3 + \mu\delta M \quad (11)$$

With Supplementary Eq. 1, we have

$$\delta M = \frac{l_2 l_3}{\Omega}\delta l_1 + \frac{l_1 l_3}{\Omega}\delta l_2 + \frac{l_1 l_2}{\Omega}\delta l_3 \quad (12)$$

With Supplementary Eq. 11-12, we have

$$\delta u = \left(P_1 + \mu\frac{l_2 l_3}{\Omega}\right)\delta l_1 + \left(P_2 + \mu\frac{l_1 l_3}{\Omega}\right)\delta l_2 + \left(P_3 + \mu\frac{l_1 l_2}{\Omega}\right)\delta l_3 \quad (13)$$

Considering that $\sigma_1 = \frac{P_1}{l_2 l_3}$, $\sigma_2 = \frac{P_2}{l_1 l_3}$, and $\sigma_3 = \frac{P_3}{l_1 l_2}$, where σ_1, σ_2, and σ_3 are three principal true stresses, we have

$$\delta u = \left(\sigma_1 + \frac{\mu}{\Omega}\right)l_d^3\lambda_2\lambda_3\delta\lambda_1 + \left(\sigma_2 + \frac{\mu}{\Omega}\right)l_d^3\lambda_1\lambda_3\delta\lambda_2 + \left(\sigma_3 + \frac{\mu}{\Omega}\right)l_d^3\lambda_1\lambda_2\delta\lambda_3 \quad (14)$$

Due to fixed Λ_α at the current state, we also have

$$\delta u = \frac{\partial u}{\partial\lambda_1}\delta\lambda_1 + \frac{\partial u}{\partial\lambda_2}\delta\lambda_2 + \frac{\partial u}{\partial\lambda_3}\delta\lambda_3 \quad (15)$$

Combing Supplementary Eq. 14 with Supplementary Eq. 15 yields

$$\left[\frac{\partial u}{\partial\lambda_1} - \left(\sigma_1 + \frac{\mu}{\Omega}\right)l_d^3\lambda_2\lambda_3\right]\delta\lambda_1 + \left[\frac{\partial u}{\partial\lambda_2} - \left(\sigma_2 + \frac{\mu}{\Omega}\right)l_d^3\lambda_1\lambda_3\right]\delta\lambda_2 + \left[\frac{\partial u}{\partial\lambda_3} - \left(\sigma_3 + \frac{\mu}{\Omega}\right)l_d^3\lambda_1\lambda_2\right]\delta\lambda_3 = 0 \quad (16)$$

Let $\delta U = \frac{\delta u}{l_d^3}$. With Supplementary Eq. 16, we have

$$\left[\frac{\partial U}{\partial\lambda_1} - \left(\sigma_1 + \frac{\mu}{\Omega}\right)\lambda_2\lambda_3\right]\delta\lambda_1 + \left[\frac{\partial U}{\partial\lambda_2} - \left(\sigma_2 + \frac{\mu}{\Omega}\right)\lambda_1\lambda_3\right]\delta\lambda_2 + \left[\frac{\partial U}{\partial\lambda_3} - \left(\sigma_3 + \frac{\mu}{\Omega}\right)\lambda_1\lambda_2\right]\delta\lambda_3 = 0 \quad (17)$$

Since $\delta\lambda_i$, i =1, 2, and 3, in Supplementary Eq. 17 are arbitrary and independent variables, we have

$$\sigma_1 = \frac{1}{\lambda_2\lambda_3}\frac{\partial U}{\partial \lambda_1} - \frac{\mu}{\Omega}$$
$$\sigma_2 = \frac{1}{\lambda_1\lambda_3}\frac{\partial U}{\partial \lambda_2} - \frac{\mu}{\Omega}$$
$$\sigma_3 = \frac{1}{\lambda_1\lambda_2}\frac{\partial U}{\partial \lambda_3} - \frac{\mu}{\Omega}. \qquad (18)$$

The change in U due to $\delta\lambda_i$ of the RVE is the sum of the change in the elastic energy of protein chains, denoted as U_1, that in the elastic energy of arms of linker proteins, denoted as U_2 , and that in the energy of mixing water with polymers, denoted as U_3, i.e.,

$$\delta U = \delta U_1 + \delta U_2 + \delta U_3. \qquad (19)$$

Among them,

$$\delta U_1 = N\delta\varepsilon^{ch}, \qquad (20)$$

where N is the number of chains per unit reference volume and ε^{ch} is the elastic energy of a single protein chain at the current state, given by

$$\begin{aligned}\varepsilon^{ch} &= \int_0^{(\lambda^{ch}-1)L_0^{ch}} F dx \\ &= \frac{k_B T}{\xi}\int_0^{(\lambda^{ch}-1)L_0^{ch}} \left[\frac{1}{4}\left(1-\frac{x}{L_c}\right)^{-2} - \frac{1}{4} + \frac{x}{L_c}\right]dx \\ &= \frac{k_B T}{\xi}\left[\frac{1}{4}L_c\left(1-\frac{(\lambda^{ch}-1)L_0^{ch}}{L_c}\right)^{-1} - \frac{(\lambda^{ch}-1)L_0^{ch}}{4} + \frac{(\lambda^{ch}-1)^2 L_0^{ch2}}{2L_c} - \frac{1}{4}L_c\right]\end{aligned} \qquad (21)$$

Since two arms of linker proteins in series behaves as a linear spring, we have

$$U_2 = \frac{1}{2}Nk^{cl}\left[(\lambda^{cl}-1)L_0^{cl}\right]^2. \qquad (22)$$

In the theory, we regard that the energy of mixing water with polymers is given by [21,22]

$$U_3 = k_B T\left[C\log\frac{\Omega C}{1+\Omega C}+\frac{\chi C}{1+\Omega C}\right], \qquad (23)$$

where χ is a measure of the interaction between polymer and water.

With Supplementary Eq. 18-23, we have

$$\sigma_1 = \frac{vN\lambda_1^2}{\lambda_1\lambda_2\lambda_3}\frac{\sqrt{3}}{6\lambda}l_d F + \frac{k_B T}{\Omega}\left[\log\left(1-\frac{1}{\lambda_1\lambda_2\lambda_3}\right)+\frac{1}{\lambda_1\lambda_2\lambda_3}+\frac{\chi}{\lambda_1^2\lambda_2^2\lambda_3^2}\right]-\frac{\mu}{\Omega}$$
$$\sigma_2 = \frac{vN\lambda_2^2}{\lambda_1\lambda_2\lambda_3}\frac{\sqrt{3}}{6\lambda}l_d F + \frac{k_B T}{\Omega}\left[\log\left(1-\frac{1}{\lambda_1\lambda_2\lambda_3}\right)+\frac{1}{\lambda_1\lambda_2\lambda_3}+\frac{\chi}{\lambda_1^2\lambda_2^2\lambda_3^2}\right]-\frac{\mu}{\Omega}$$
$$\sigma_3 = \frac{vN\lambda_3^2}{\lambda_1\lambda_2\lambda_3}\frac{\sqrt{3}}{6\lambda}l_d F + \frac{k_B T}{\Omega}\left[\log\left(1-\frac{1}{\lambda_1\lambda_2\lambda_3}\right)+\frac{1}{\lambda_1\lambda_2\lambda_3}+\frac{\chi}{\lambda_1^2\lambda_2^2\lambda_3^2}\right]-\frac{\mu}{\Omega}. \qquad (24)$$

In the theory, the free swelling state is considered to be the initial state, which is at a steady state. At this state, $\sigma_1 = \sigma_2 = \sigma_3 = 0$. Meanwhile, with Supplementary Eq. 7, the fraction of unfolded domains at the free swelling state, denoted as Λ_α^s, is given by

$$\Lambda_\alpha^s = \frac{1}{1+k_{\alpha 0}\exp\left(-\frac{F_0 \Delta z_\alpha}{k_B T}\right)}, \qquad (25)$$

where$k_{\alpha 0} = k_{\alpha 0}^{f}/k_{\alpha 0}^{uf}$,$\Delta z_\alpha = \Delta z_\alpha^{f} + \Delta z_\alpha^{uf}$ and F_0 is the chain force at the free swelling state. As shown in Figure 3b, circular rings made of gels were loaded by two metallic hooks in experiments. Since gels were very soft and these rings were easily to be bended, very small loads were required to deform rings to a straight configuration in experiments, as shown in the middle of Figure 3b. This straight configuration was then taken as the initial configuration in our experiments. Beyond this point, the hydrogels were regarded to be under uni-axial tension, as seen on the right of Figure 3b. Correspondingly in our theory, we modeled gels with a representative volume element under uniaxial tension along "1" direction with $\sigma_2 = \sigma_3 = 0$, as illustrated in Supplementary Figure 15a. However, strictly speaking, the loading condition in the

ring samples is not exactly uniaxial tension in our experiments, as suggested by our simulation results of a hoop sample (with a neo-hookean constitutive relation) using ABAQUS (Supplementary Figure 15b-e). It is expected that the approximation of uni-axial tension to simplify the calculation in our theory should not affect the theoretical results in any significant ways. It would also be great to improve the theoretical calculation in our future work.

To compare with experiments, gels are considered to be subjected to uniaxial loading/unloading at a constant rate, $\gamma = \frac{\Delta L}{L_0 \Delta t}$, where L_0 is the length of the hydrogel at the free swelling state, ΔL the length change within a time interval of Δt. In the numerical analysis, an explicit method is employed. Default parameters in the analysis for Gel-1, Gel-2, and Gel-3 are listed in Supplementary Tables 10-12, respectively. The state variables at the free swelling state, such as Λ_a^s and the principal stretch, denoted as λ^s, are firstly determined with Supplementary Eq. 24-25, which are set as initial conditions. In Figure 4c-e, the stress is the nominal tensile stress along "1" direction, denoted as s_1, given by $s_1 = \sigma_1 \lambda_2 \lambda_3 / \lambda^{s2}$, and the strain is given by $\lambda_2/\lambda^s - 1$ with the load being applied along "1" direction. For Gel-1 or Gel-3, only one type of domain is considered in the analysis.

The failure of Gel-1, Gel, 2 or Gel-3 in the experiments upon mechanical loadings is due to fracture. According to the fracture theory[23], the failure stress, denoted as σ_f, is given by

$$\sigma_f = \sqrt{\frac{EG}{\pi a}}, \qquad (26)$$

where E is taken as the initial elastic modulus, G the fracture toughness, and a the crack size. Considering that the fracture toughness of gels, denoted as G, is due to the rupture of the protein chains lying across the path of the crack[24],

$$G = \varpi N^{2/3}, \qquad (27)$$

where ϖ is the energy required to rupture a single protein chain and $N^{2/3}$ corresponds to area density of protein chains. Note that protein chains are initially subjected to a pre-stretch at the free swelling state of gels. The final rupture of a single protein chain is due to bond breaking of one arm of linker proteins that is in series with the protein chain instead. Due to the nature of multiple bonds existing within gels, the apparent bond breaking force of a crosslinker should also depend on its rebinding kinetics. In calculating ϖ, a protein chain is subjected to a constant stretching velocity, 10 nm s^{-1} and the apparent bond breaking force is set to be 20 pN, based on the loading rate dependent experiments for the rupture forces shown in Extended Data Figure 1. ϖ is found to be 92.3 zJ for Gel-1, 464 zJ for Gel-2, and 977 zJ for Gel-3, respectively. With Supplementary Eq. 26-27, the ratio among failure stresses for Gel-1, Gel-2, and Gel-3 is calculated to be 1.4:1:2, which is close to the experimental value, 1:1:2, shown in Figs. 3c-d. The crack size in gels is also estimated, which is on the order of 0.5 μm.

The RVE used in our theory was essentially similar to the 8-chain model[17], which was a classical network model to describe mechanical properties of polymeric materials. The 8-chain model[17] was mathematically simple but also able to capture various deformation modes, including uniaxial tension, pure shear, and biaxial tension. By now, the 8-chain model[17] has also been incorporated into various theories to describe gel deformation[25,26]. Other polymeric models, such as 3-chain model[27], 4-chain model[28,29], or full network model[30] may also model our system well and the similar trends can be expected. The 8-chain model we used can capture shear deformation well, as documented in literature[31].

Among parameters adopted in our simulation as listed in Supplementary Tables 10-12, ξ, Ω, L_0^{cl}, k^{cl}, γ, χ, μ were the same for Gel-1, Gel-2, and Gel-3. The unfolding rate, the unfolding distance, the refolding rate, or the refolding distance for different domains within LBMs for Gel-1, Gel-2,

or Gel-3 were different and taken from experiments, as listed in Supplementary Table 1. The change in contour length when a domain was unfolded was different for different domains and obtained by subtracting respective folded length from respective unfolded length listed in Supplementary Table 1. Other parameters adopted in our simulation, as listed in Supplementary Tables (10-12), are also given in Supplementary Table 13 for better comparison. It is expected that all these parameters would affect simulated rigidity, toughness and extensibility in our theory.

The comparison between theory and experiments

The comparison of Young's modulus, failure strain, and fracture toughness between theory and experiment was provided in Supplementary Tables 14-16, respectively. Note that the fracture toughness from experiments was obtained through Eq. (S26) with measured E and σ_f from experiments and also an assumption of a crack size of 0.5 μm. As seen from Supplementary Tables 14-16, there exist mismatches between experiment and theory.

In our theory, it was regarded that proteins gels form a crosslinked three-dimensional network with its mechanical properties described by RVE shown in Fig. 3b. However, in reality, the crosslinked protein network within fabricated gels might not be so perfect as that illustrated in Figure 1a. For example, some arms of CLs might not be able to form crosslinks with LBMs and some LBMs might have just hanged to the network from one of its end or both ends might be separated from the network. Due to these defects, protein length in the main network may be inhomogeneous and unfolding or re-folding dynamics of folded domains with LBMs would be affected, which are expected to affect mechanical properties of fabricated gels in turn. In addition, the loading condition was assumed to be uniaxial tension in the simulation, which is slightly different from that in our experiments. These might be part of the reasons why the shapes of hysteresis obtained

from experiments look different from our theoretical prediction, as comparing Figure 3 with Figure 4.

It should be pointed out that the crack size, denoted as a in Eq. (S26), was not determined. We therefore suggest that the discrepancy of facture toughness between theory and experiment might be due to the different crack sizes existing within three different gels. Nevertheless, defects in the crosslinked protein network, voids, and other damages, as well as their evolution under loads might also have contributed this discrepancy.

To improve our theory further, inhomogeneity of protein network of fabricated gels may be captured by varying the length, number of domains, or unfolding or re-folding dynamics of domains, etc. of 8 chains within a RVE. To better predict fracture toughness, it is desirable to find out initial defects within gels in experiments. These defects should then be incorporated into a fracture theory, which also considers how these defects evolve. In addition, the breaking and reformation of bonds between LBMs and CLs have not considered in our theory yet, which should be included in predicting the healing process of gels in our future work. It would also be great to simulate the load-displacement curve of hoop samples, as used in the experiments, by incorporating the constitutive relation developed in our work into a program for finite element method, such as ABAQUS.

Statistical analysis

Igor Pro and Microsoft Excel were used to plot and analyze all the graphs. Student's t test was used for statistical analyses. When P value is below 0.05, it is considered being statistically significant.

Supplementary References

1 Cao, Y. & Li, H. Polyprotein of GB1 is an ideal artificial elastomeric protein. *Nat Mater* **6**, 109-114, doi:10.1038/nmat1825 (2007).

2 Zoldak, G., Stigler, J., Pelz, B., Li, H. & Rief, M. Ultrafast folding kinetics and cooperativity of villin headpiece in single-molecule force spectroscopy. *Proc Natl Acad Sci U S A* **110**, 18156-18161, doi:10.1073/pnas.1311495110 (2013).

3 Jagannathan, B., Elms, P. J., Bustamante, C. & Marqusee, S. Direct observation of a force-induced switch in the anisotropic mechanical unfolding pathway of a protein. *Proc Natl Acad Sci U S A* **109**, 17820-17825, doi:10.1073/pnas.1201800109 (2012).

4 Schoeler, C. *et al.* Ultrastable cellulosome-adhesion complex tightens under load. *Nat Commun* **5**, 5635, doi:10.1038/ncomms6635 (2014).

5 Li, Y. D., Lamour, G., Gsponer, J., Zheng, P. & Li, H. The molecular mechanism underlying mechanical anisotropy of the protein GB1. *Biophys J* **103**, 2361-2368 (2012).

6 Cai, S. & Suo, Z. Mechanics and chemical thermodynamics of phase transition in temperature-sensitive hydrogels. *J Mech Phys Solids* **59**, 2259-2278, doi:10.1016/j.jmps.2011.08.008 (2011).

7 Lv, S. *et al.* Designed biomaterials to mimic the mechanical properties of muscles. *Nature* **465**, 69-73, doi:10.1038/nature09024 (2010).

8 Lv, C. *et al.* Low folding cooperativity of HP35 revealed by single-molecule force spectroscopy and molecular dynamics simulation. *Biophys J* **102**, 1944-1951, doi:10.1016/j.bpj.2012.03.028 (2012).

9 Cao, Y. & Li, H. Engineering tandem modular protein based reversible hydrogels. *Chem Commun (Camb)*, 4144-4146, doi:10.1039/b806684a (2008).

10 Li, Y. *et al.* Single-molecule study of the synergistic effects of positive charges and Dopa for wet adhesion. *J. Mater. Chem. B*, doi:10.1039/c7tb00131b (2017).

11 Li, Y. *et al.* Single molecule force spectroscopy revealing multiple binding modes between DOPA and different rutile surfaces. *CHEMPHYSCHEM*, doi:10.1002/cphc.201600374 (2016).

12 Li, Y., Qin, M., Li, Y., Cao, Y. & Wang, W. Single molecule evidence for the adaptive binding of DOPA to different wet surfaces. *Langmuir* **30**, 4358-4366, doi:10.1021/la501189n (2014).

13 Huang, W., Qin, M., Li, Y., Cao, Y. & Wang, W. Dimerization of Cell-Adhesion Molecules Can Increase Their Binding Strength. *Langmuir* **33**, 1398-1404, doi:10.1021/acs.langmuir.6b04396 (2017).

14 Yan, X. *et al.* Molecular mechanism of inward rectifier potassium channel 2.3 regulation by tax-interacting protein-1. *J Mol Biol* **392**, 967-976, doi:10.1016/j.jmb.2009.07.060 (2009).

15 Zhang, X. *et al.* Rational design of a tetrameric protein to enhance interactions between self-assembled fibers gives molecular hydrogels. *Angew Chem Int Ed Engl* **51**, 4388-4392, doi:10.1002/anie.201108612 (2012).

16 Ito, F. *et al.* Reversible hydrogel formation driven by protein-peptide-specific interaction and chondrocyte entrapment. *Biomaterials* **31**, 58-66, doi:10.1016/j.biomaterials.2009.09.026 (2010).

17 Arruda, E. M. & Boyce, M. C. A three-dimensional constitutive model for the large stretch behavior of rubber-elastic materials. *J Mech Phys Solids* **41**, 389-412, doi:10.1016/0022-5096(93)90013-6 (1993).
18 Bell, G. I. Models for the specific adhesion of cells to cells. *Science* **200**, 618-627 (1978).
19 Rief, M., Fernandez, J. M. & Gaub, H. E. Elastically coupled two-level systems as a model for biopolymer extensibility. *Phys Rev Lett* **81**, 4764-4767, doi:10.1103/PhysRevLett.81.4764 (1998).
20 Marko, J. F. & Siggia, E. D. Stretching dna. *Macromolecules* **28**, 8759-8770 (1995).
21 Huggins, M. L. Solutions of long chain compounds. *J Chem Phys* **9**, 440-440, doi:10.1063/1.1750930 (1941).
22 Flory, P. J. Thermodynamics of high polymer solutions. *J Chem Phys* **10**, 51-61, doi:10.1063/1.1723621 (1942).
23 Lawn, B. R. *Fracture of brittle solids.* 2nd edn, (Cambridge Solid State Science Series, 1993).
24 Lake, G. J. & Thomas, A. G. The strength of highly elastic materials. *Proc. Roy. Soc. (Lond.) A* **300**, 108 (1967).
25 Lopez-Menendez, H. & Rodriguez, J. F. Microstructural model for cyclic hardening in F-actin networks crosslinked by alpha-actinin. *J Mech Phys Solids* **91**, 28-39, doi:10.1016/j.jmps.2016.01.015 (2016).
26 Wang, Q. M. & Gao, Z. M. A constitutive model of nanocomposite hydrogels with nanoparticle crosslinkers. *J Mech Phys Solids* **94**, 127-147, doi:10.1016/j.jmps.2016.04.011 (2016).
27 Wang, M. C. & Guth, E. Statistical Theory of Networks of Non-Gaussian Flexible Chains. *J Chem Phys* **20**, 1144-1157, doi:Doi 10.1063/1.1700682 (1952).
28 Flory, P. J. & Rehner, J. Statistical mechanics of cross-linked polymer networks I Rubberlike elasticity. *J Chem Phys* **11**, 512-520, doi:Doi 10.1063/1.1723791 (1943).
29 Treloar, L. R. G. The Elasticity of a Network of Long-Chain Molecules .3. *T Faraday Soc* **42**, 83-94, doi:Doi 10.1039/Tf9464200083 (1946).
30 Wu, P. D. & Vandergiessen, E. On Improved Network Models for Rubber Elasticity and Their Applications to Orientation Hardening in Glassy-Polymers. *J Mech Phys Solids* **41**, 427-456, doi:Doi 10.1016/0022-5096(93)90043-F (1993).
31 Boyce, M. C. & Arruda, E. M. Constitutive models of rubber elasticity: A review. *Rubber Chem Technol* **73**, 504-523, doi:Doi 10.5254/1.3547602 (2000).
